# Unique Lipid Signatures of Extracellular Vesicles from the Airways of Asthmatics

**DOI:** 10.1038/s41598-018-28655-9

**Published:** 2018-07-09

**Authors:** Kenneth P. Hough, Landon S. Wilson, Jennifer L. Trevor, John G. Strenkowski, Njeri Maina, Young-Il Kim, Marion L. Spell, Yong Wang, Diptiman Chanda, Jose Rodriguez Dager, Nirmal S. Sharma, Miranda Curtiss, Veena B. Antony, Mark T. Dransfield, David D. Chaplin, Chad Steele, Stephen Barnes, Steven R. Duncan, Jeevan K. Prasain, Victor J. Thannickal, Jessy S. Deshane

**Affiliations:** 1 Department of Medicine, Division of Pulmonary, Allergy and Critical Care Medicine, University of Alabama at Birmingham, Birmingham, AL USA; 2 Center for AIDS Research, University of Alabama at Birmingham, Birmingham, AL USA; 3 Department of Microbiology, University of Alabama at Birmingham, Birmingham, AL USA; 4 Department of Pharmacology and Toxicology, University of Alabama at Birmingham, Birmingham, AL USA; 5 Targeted Metabolomics and Proteomics Laboratory, University of Alabama at Birmingham, Birmingham, AL USA

## Abstract

Asthma is a chronic inflammatory disease process involving the conductive airways of the human lung. The dysregulated inflammatory response in this disease process may involve multiple cell-cell interactions mediated by signaling molecules, including lipid mediators. Extracellular vesicles (EVs) are lipid membrane particles that are now recognized as critical mediators of cell-cell communication. Here, we compared the lipid composition and presence of specific lipid mediators in airway EVs purified from the bronchoalveolar lavage (BAL) fluid of healthy controls and asthmatic subjects with and without second-hand smoke (SHS) exposure. Airway exosome concentrations were increased in asthmatics, and correlated with blood eosinophilia and serum IgE levels. Frequencies of HLA-DR^+^ and CD54^+^ exosomes were also significantly higher in asthmatics. Lipidomics analysis revealed that phosphatidylglycerol, ceramide-phosphates, and ceramides were significantly reduced in exosomes from asthmatics compared to the non-exposed control groups. Sphingomyelin 34:1 was more abundant in exosomes of SHS-exposed asthmatics compared to healthy controls. Our results suggest that chronic airway inflammation may be driven by alterations in the composition of lipid mediators within airway EVs of human subjects with asthma.

## Introduction

Asthma is a chronic inflammatory disease of the airways that afflicts over 300 million people worldwide^1–3^. The immunological basis of asthma is complex^4^, and involves immune cell-epithelial cross-talk^5^. Environmental antigens are presented by antigen presenting cells to activate CD4^+^ T cells, promoting their proliferation and production of T helper cytokines that drive inflammatory responses^6^. Moreover, exposures to cigarette smoke not only negatively influence innate immune responses, but enhance inflammatory cell signaling that predispose asthmatics to exacerbations^7,8^.

Pro-inflammatory lipids have also been implicated in the pathogenesis of asthma^9–19^. Lipid mediators drive airway inflammation, promote immune cell infiltration, and induce mucous hyperplasia. Lipids such as ceramides, prostaglandins, and leukotrienes are potent mediators of inflammation in the airways of asthmatics^13,14,20,21^. Asthmatics have much higher concentration of leukotrienes in their exhaled breath condensate and sputum as compared to healthy controls^15,19^. In general, these lipids are rapidly generated at the site of inflammation by myeloid-lineage and other airway cells^22–24^ and drive the recruitment of Th2 cells into the lungs, mediating the classical Th2-mediated inflammatory response^25^. Ceramides, sphingomyelins, and phospholipids are involved in cellular survival and apoptosis, airway remodeling, and immune cell activation^26–33^. Furthermore, the balance between the levels of ceramides and sphingomyelins has been shown to be critical in regulating cell survival, differentiation, and apoptosis, thus leading to the concept of the sphingolipid rheostat^34–36^.

Extracellular vesicles (EVs) have recently been reported to participate in the inflammatory responses accompanying chronic lung diseases^37–43^. EVs are membrane bound particles that encompass both endosome-derived exosomes enriched in tetraspanins and microparticles derived from cell membrane^44–48^. EVs facilitate intercellular communication and may promote chronic inflammation by recruiting immune cells^39,42,49–54^ and have been implicated in disease pathogenesis and cell-cell signaling^39,40,52,55–58^. EVs contain bioactive cargo, such as proteins, nucleic acids (such as microRNA, lnRNA, and mRNA), and lipids^59,60^. For example, bioactive lipids contained in platelet-derived EVs can mediate transcellular activation of platelets and endothelial cells^61^. Reciprocally, endothelial cell derived EVs activate neighboring pericytes via pro-inflammatory microRNAs^62^. Stromal cell-derived exosomes have been shown to modulate the polarization of macrophages^63^. Furthermore, the lipid cargo of exosomes has been implicated in altering cellular function and signaling^58,64^. Ceramides, sphingomyelins, phosphatidylserines and phosphatidylcholines are known lipid constituents of EVs^60^. For example, ceramides in exosomes induce apoptosis in oligodendroglioma cells^65^, and promote macrophage chemotaxis in a mouse model^53^. Furthermore, ceramides play an important role in budding of exosomes^66^. Enzymes capable of producing leukotrienes, potent lipid mediators that promote granulocyte migration, were identified in exosomes generated from healthy human monocyte dendritic cells and monocyte-derived macrophages^39^, as well as in epithelial cancer cells from the lung^58^.

Previous reports indicate that EVs from the bronchoalveolar lavage (BAL) fluid of asthmatics express higher levels of class-II molecules, in particular HLA-DR, and adhesion molecules such as ICAM-1 (also known as CD54)^28,50,52^. HLA-DR is a class-II antigen presentation molecule on antigen presenting cells, which is important in activation of lymphocytes, and is associated with asthma pathogenesis^67^. It has also been shown to be expressed on activated bronchial epithelial cells of asthmatics, along with elevated ICAM-1 expression^68^. Active pro-inflammatory mediators and cytokines have also been identified in exosomes from asthmatic airways^52,69^. Although the role of EVs in asthma is just beginning to be elucidated, the lipid signatures of airway EVs that may contribute to chronic inflammation have not been described in the context of asthma. Additionally, understanding how environmental exposures may impact lipid mediated-inflammation by EVs in asthma will provide new insights for developing new therapeutic strategies targeting lipid mediators. In this study, we characterize for the first time, the lipid composition of EVs from asthmatics and healthy subjects, as well as secondhand smoke (SHS) exposed subjects from each group. We have identified significant differences in the abundance of ceramides, modified ceramides, and phosphatidylglycerol in airway EVs of asthmatics and their alterations in SHS-exposed individuals. Our findings provide new insights on how EVs may drive inflammatory responses and exacerbate inflammation in SHS-exposed asthmatics.

## Methods

### Study Design and Subjects

Asthmatic and healthy control subjects were enrolled through the University of Alabama at Birmingham Lung Health Center following the inclusion and exclusion criteria, and screened for IgE titer, blood eosinophil frequencies, past medical history, and FEV_1_. Healthy controls did not have histories of asthma, pulmonary infections or other known lung diseases. All asthmatic patients had a prior diagnosis of asthma and demonstrated a 12% or greater increase in FEV_1_ within 30 minutes of administrating 400 μg of albuterol, as outlined in the GINA guidelines (Global Strategy for Asthma Management and Prevention; http://www.ginasthma.org/)^70^. Screened subjects with serum cotinine levels greater than 10 ng/ml and those subjects who received treatment with inhaled or systemic corticosteroids within the six weeks prior to or during the study were excluded. Previous exposure to secondhand smoke was determined by LC-MRM mass spectrometry for serum cotinine levels between 0.05–10 ng/ml^71,72^. Healthy controls and asthmatics with serum cotinine levels below 0.05 ng/ml were considered non-SHS-exposed subjects.

Bronchoscopies were carried out as previously described^73,74^. In brief, a total of 200 ml of saline solution was instilled, with an average return of 108.8 ± 18.8 ml among healthy, and 101.9 ± 36.6 ml for asthmatics. Forty (40 ml) of BAL fluid was used for isolation of extracellular vesicles (EVs). From the purified EVs, 1 × 10^9^ particles were used for lipidomics analysis, and the remaining for characterization of EVs. The study was approved by the University of Alabama at Birmingham Institutional Review Board (Protocol #F151209005), and written informed consent was obtained from all participants. All procedures performed in this study were performed in accordance with relevant guidelines and regulations. Study subjects were recruited for a study funded by the Flight Attendant Medical Research Institute (FAMRI).

### Isolation of Extracellular Vesicles from BAL Fluid

Extracellular vesicles were isolated using a previously described differential centrifugation method^75^. Briefly, 40 ml of BAL fluid was centrifuged at 300 × g for 10 minutes at 4 °C to remove airway cells. Then the supernatant was further centrifuged at 2,000 × g for 10 minutes at 4 °C to remove any dead cells and large cellular debris. The supernatant was spun again at 10,000 × g for 30 minutes at 4 °C and filtered through a 0.2 μm cellulose acetate filter (Corning, New York). The filtrate was then centrifuged at 100,000 × g for 70 minutes at 4 °C and the pellet washed with fresh PBS to remove any contaminating proteins. Finally, the washed pellet was centrifuged at 100,000 × g for 70 minutes at 4 °C, and the pellet resuspended in 100 µl of fresh PBS. The purified EVs were stored at −80 °C.

### Quantitation of EVs

The concentration and size distributions of purified airway EVs were determined using a NanoSight NS300 (Cambridge, MA). The instrument was calibrated using 100 nm polystyrene latex microspheres (Malvern Instruments Ltd., Malvern, UK). EVs were diluted 1,000-fold with PBS to make a final volume of 1 ml and loaded in to a 1 ml syringe, which was placed on a syringe pump attached to the NanoSight. The diluted EVs were injected into the NanoSight at a flow rate of 25 μl/s at room temperature. A total of 5 videos were acquired per sample under the following conditions and settings: temperature: 22.4–22.6 °C; viscosity 0.939–0.944 cP; camera level: 7; capture duration: 1 min/video; shutter speed: 11.12 ms; camera type: SCMOS; gain: 1; minimum tracks completed: 2000–4000/video; frames processed: 1951/video; frames per second: 32.5 fps; blur: auto; and detection threshold: 5.

### Electron Microscopy of EVs

For electron microscopy analysis, 10 μl of undiluted airway EVs were stained negatively and imaged on a FEI Tecnai T12 (Hillsboro, Oregon) electron microscope at the UAB High Resolution Imaging Facility. The EVs were imaged at 104,000 × magnification.

### ImageStream Analysis of EVs

ImageStream combines flow cytometry with fluorescence imaging technology, and can resolve much smaller particles than conventional flow cytometry. CD63 eFlour450 (clone H5C6; Affymetrix, Inc., Santa Clara) was used as a positive marker to distinguish exosomes from other types of EVs, cellular debris, and calibration beads. EVs were also stained with antibodies to HLA-DR APC (clone LN3; Affymetrix, Inc., Santa Clara), CD54 PE (clone HA58; Affymetrix, Inc., Santa Clara), CD9 PE (clone M-L13; BD Biosciences, San Jose, CA), CD81 PE-Cy7 (clone 5A6; BioLegend, Inc., San Diego, CA), Grp94 DyLight 488 (clone 9G10; Enzo Life Sciences, Inc., Framingdale, NY), and ARF6 APC (AssayPro, LLC, St. Charles, MO). For the PKH26 (Sigma, St. Louis, MO) and CellTrace CFSE (Invitrogen, Carlsbad, CA) experiments, EVs were labeled with their respective dyes following the manufacturer’s protocols. CFSE labeling was stopped with 3% BSA in PBS instead of FBS, which contains exogenous EVs, such as exosomes. The stained samples were imaged at 60 × magnification with extended depth of field (EDF), while acquiring data on channels Ch01, Ch03, Ch06, Ch07, Ch09, Ch11 and Ch12. Appropriate controls, single color stains, and calibration beads were used to adjust spectral compensation and to calibrate the machine. A total of 5,000 events were acquired. Channels Ch01 and Ch09 were used as brightfields, and Ch12 was used for side-scatter. The acquired data was analyzed using IDEAS software version 6.2 (EMD Millipore, Bellerica, MA).

### Flow Cytometry Analysis of EVs

A total of 1 × 10^7^ particles of airway EVs were stained with antibodies specific for CD63, CD54, HLA-DR, CD9, CD81, ARF6, Grp94, TSG101 Alexa Fluor 647 (clone 4A10; Novus Biologicals, Littleton, CO), and CD36 FITC (clone eBioNL07). Flow cytometry data was acquired on a BD Becton Dickinson LSRII (Franklin Lakes, NJ). This machine was configured to detect small particles by changing the photomultiplier tube (PMT) voltage for forward and side scatters to 600 volts and 286 volts respectively, and thresholding on both forward and side scatters to 500 volts. ApogeeBead Mix (Apogee Flow Systems Ltd., Hertfordshire, UK), were used to determine approximate gating location of EVs based on size on a forward and side scatter plot. Particles between 50–150 nm were gated and total of 100,000 events were acquired. PBS with antibodies alone was used as a control to confirm the absence of any potential background signals from free, unbound antibodies in the samples. Additionally, sheath fluid from the fluidics system of the LSRII to determine noise or interfering particles that may hinder EV flow data acquisition. Fluorescence compensation was configured using single color stained samples of EVs. Acquired flow cytometry data was analyzed using FlowJo X.

### Sample Preparation and Extraction of Lipids for Lipidomics Analyses

Untargeted analysis for lipids was performed on 1 × 10^9^ particles of EVs isolated from the BAL fluid obtained from asthmatics, and healthy subjects. Patients were categorized based on disease status, as well as prior exposure to secondhand smoke (SHS). Lipids were extracted from EVs using methanol:chloroform (2:1). Additionally, a C17 ceramide internal standard was used at a concentration of 10 ng/ml per sample added to the methanol component of the extraction procedure. Briefly, samples were treated with 100 μl of 6 N HCl in a glass tube and vortexed for 15 s, before extraction. Sample mixture was incubated on ice for 10 minutes. 1 ml of chloroform was added followed by 1 ml of purified water, and vortexed for 15 seconds. The sample was centrifuged at 3000 RPM at 10 °C for 10 minutes in a Beckman GS-6R Centrifuge. The lower, organic phase, was transferred to a new glass tube. The extraction procedure was repeated by adding 1 ml of chloroform to the remaining upper phases, vortexing for 15 s and centrifuging again at the same conditions. The lower, organic phase, was pooled and dried at 37 °C-40 °C under a stream of argon gas, using the Reacti-Therm III (ThermoFisher Scientific, Waltham, MA) heating module and Reacti-Vap III (ThermoFisher Scientific, Waltham, MA) evaporator.

### SWATH Lipidomics Analysis

Untargeted lipidomics was performed on dried lipids using a SCIEX 5600 (AB Sciex Pte. Ltd., Framingham, MA) TripleTof mass spectrometer in both positive and negative modes by direct infusion. The direct infusion solvent was methanol:chloroform (2:1) with 5 mM ammonium acetate, and samples were delivered to the source by isocratic flow at 7 μl/min using a 500 μl Hamilton Gas Tight Syringe. Prior to and in between samples, the syringe used for direct infusion was cleaned with 2 flushes each of 100% methanol, 100% acetonitrile, 100% isopropyl alcohol, and 100% direct infusion solvent. Calibration runs were performed in both positive and negative modes with the APCI Positive Calibration Solution and APCI Negative Calibration Solution, respectively. For both positive and negative runs, lipids were infused at a constant rate of 7 μl/min. The analyte mass evaluation range was 200–1200 *m/z*. A high resolution Tof scan was acquired initially for 250 ms, then a series of high sensitivity product ion scans were acquired per one Dalton (1 *m/z*) mass starting at 200 *m/z* through 1200 *m/z*. The collision energy was fixed at 35 eV, curtain gas to 20.00, GS1 to 20.00, GS2 to 15.00, spray voltage to 500 for positive ion mode and 4500 for negative ion mode, and temperature set to 400 °C. The entire method took 6 minutes for acquisition.

### SWATH Analysis

The acquired mass spectrometry data were processed with SCIEX LipidView 1.2 software (Concord, ON, Canada). Lipid identities were assigned by LipidView, which utilizes a database of known ion fragmentations. To confirm selected lipid identities, SCIEX PeakView 1.2 (Concord, ON, Canada) was used to further investigate ions. The mass tolerance window was set to 5 mDa and the peaks greater than a signal-to-noise ratio of 3 were considered for analysis. Identification of individual lipid species from LipidView assignments was based on mass accuracy (<5 ppm) and MS/MS spectra obtained from PeakView. High resolution TOFMS provided the accurate mass of precursor ions.

### Statistical analysis

Lipid peaks were first normalized by the mean and standard deviation value of each sample, prior to statistical analysis. Lipids with missing values were removed entirely from the analysis. All analyses were performed on 91 lipid measurements. Lipidomics data were summarized by presenting descriptive statistics along with bivariate comparisons of paired two groups that included parametric and non-parametric statistics. Graphical illustrations were generated to describe visual differences in lipids between two groups. Principal Component Analysis (PCA) and Partial Least Square Discriminant Analysis (PLS-DA) were performed to determine which lipids contribute to separation of the four groups. MetaboAnalyst 3.0 (www.metaboanalyst.ca) was utilized for data transformation and multivariate analyses (PCA and PLS-DA). Mann Whitney T-test was used for bivariate analyses. Continuous variables were analyzed by Spearman correlation. P-values of 0.05 or less were considered as significant. Statistical analyses were performed by using SAS 9.4 and GraphPad Prism5.

### Data availability

The data generated and or analyzed in this study are available from the corresponding author upon request.

## Results

### Concentrations of EVs are increased in asthmatics and correlates with plasma eosinophilia

To characterize EVs from the airways of asthmatics, we obtained BAL fluid from a cohort of asthmatic subjects and healthy controls. The characteristics of the human subjects studied here are described in Table 1. The mean age of enrolled subjects was 43 for healthy individuals, and 42 for asthmatics. Over 50% of the individuals had exposure to secondhand smoke (SHS). Statistically significant differences existed among groups in IgE titer and post-bronchodilator FEV_1_.

**Table 1:** Characteristics of enrolled study subjects. IQR, interquartile range; IgE, Immunoglobulin Epsilon; FEV_1_, forced expiratory volume in 1-second; p-values from Student’s t-test.

Characteristics of Enrolled Subjects			
Characteristics	Healthy (N = 9)	Asthmatics (N = 11)	p-value
Sex			
*Male*	1	0	
*Female*	8	11	
Median age (IQR)	43 (17.5)	42 (24.8)	0.6865
Secondhand smoke exposure	5 (50%)	6 (50%)	
Median IgE titer (IQR)	14.9 (20.1)	439 (630)	0.0007
Median post-FEV_1_ (IQR)	107 (21.5)	85 (12.0)	0.0009

**Figure 1:**
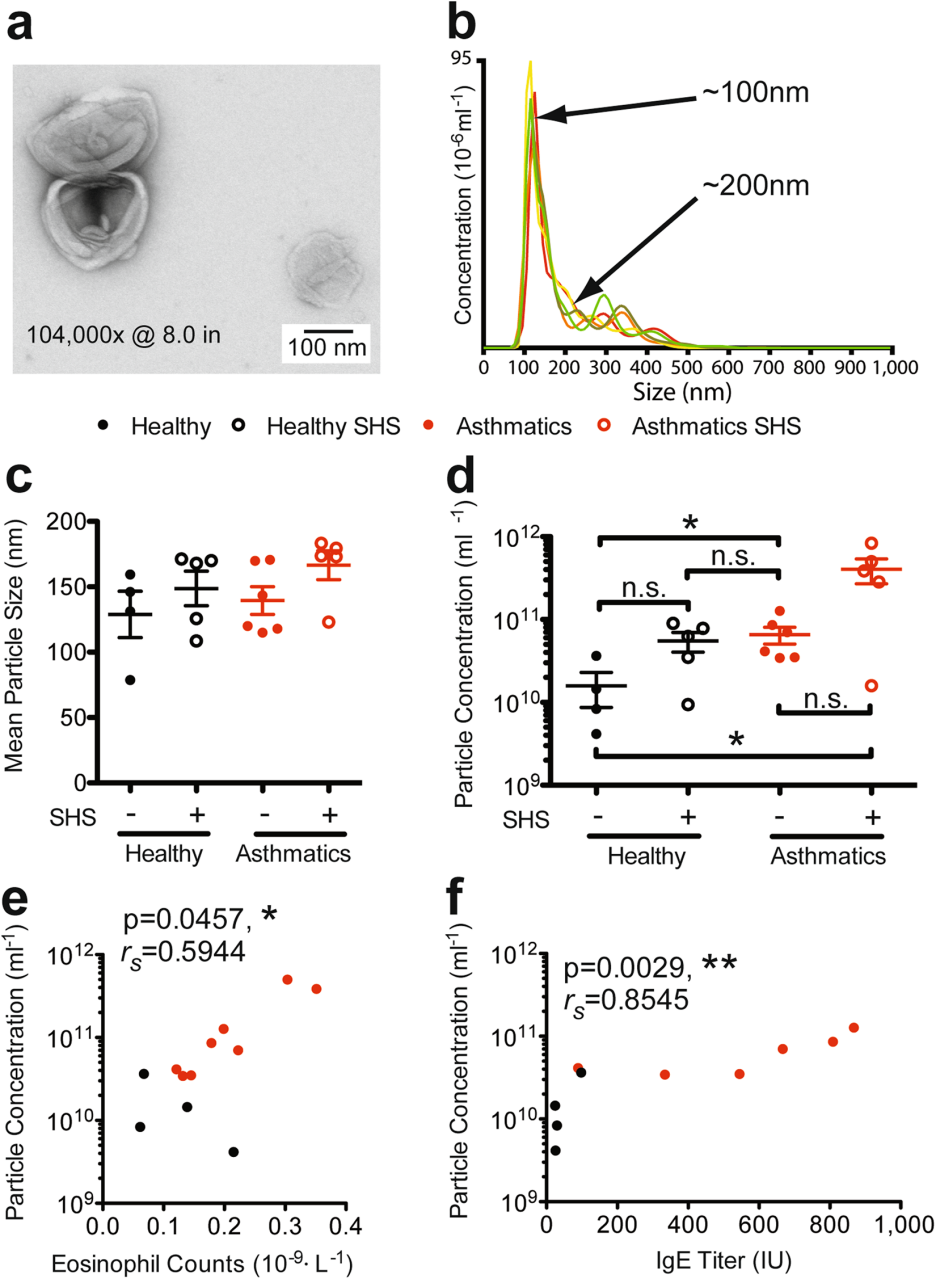
General characteristics of BAL extracellular vesicles (EVs). (**a**) Electron microscopy of negative-stained BAL EVs. (**b**) NanoSight quantitation of a representative BAL EV isolation showing concentration on the y-axis, and size distribution on the x-axis. (**c**) Comparison of size distribution of EVs between each study group. (**d**) Comparison of EV particle concentration between healthy subjects, asthmatics, and SHS-exposed groups. Mann Whitney T test, * < 0.05. (**e**) Correlation of eosinophil frequency and particle concentration. Spearman’s rank correlation, * < 0.05. (**f**) Correlation of IgE titer and particle concentration. Spearman’s rank correlation, ** < 0.01; (Healthy Subjects, n = 9; Asthmatic Subjects, n = 11).

We first confirmed the morphology of purified EVs by electron microscopy (EM). EM analysis confirmed a “deflated-football” like morphology as previously described^55^ (Fig. 1a). We then determined size distribution and concentration of purified EVs using NanoSight (Cambridge, MA). NanoSight analyses indicated that particles isolated from the BAL ranged from 50–150 nm representing the standard size range of exosomes (Fig. 1b). We compared the size distribution of EVs between asthmatics and healthy subjects. Although no statistically significant difference was observed, mean diameters for BAL EVs of asthmatics and SHS-exposed asthmatics were higher compared to healthy subjects (Fig. 1c). Particle concentration was significantly higher in both asthmatics and SHS-exposed asthmatics compared to healthy individuals with no prior SHS-exposure (Fig. 1d). We performed NanoSight analysis on pre- and post- freeze-thawed EVs to determine whether freeze-thaws could alter or damage the EV composition. No significant differences in concentration or size distribution were observed (data not shown).

We then analyzed whether EV concentration correlated with the clinical phenotype of allergic airway inflammation, characterized by peripheral eosinophilia and elevated IgE levels. EV concentrations significantly correlated with blood eosinophilia (*r*_*s*_ = 0.59, p = 0.046) (Fig. 1e), as well as with IgE titers (*r*_*s*_ = 0.85, p = 0.003) (Fig. 1f). These data support the concept that allergic inflammation is associated with increased production of EVs in asthmatic airways.

### Extracellular vesicle markers are differentially expressed on BAL EVs from asthmatics, SHS-exposed groups and their respective controls

**Figure 2:**
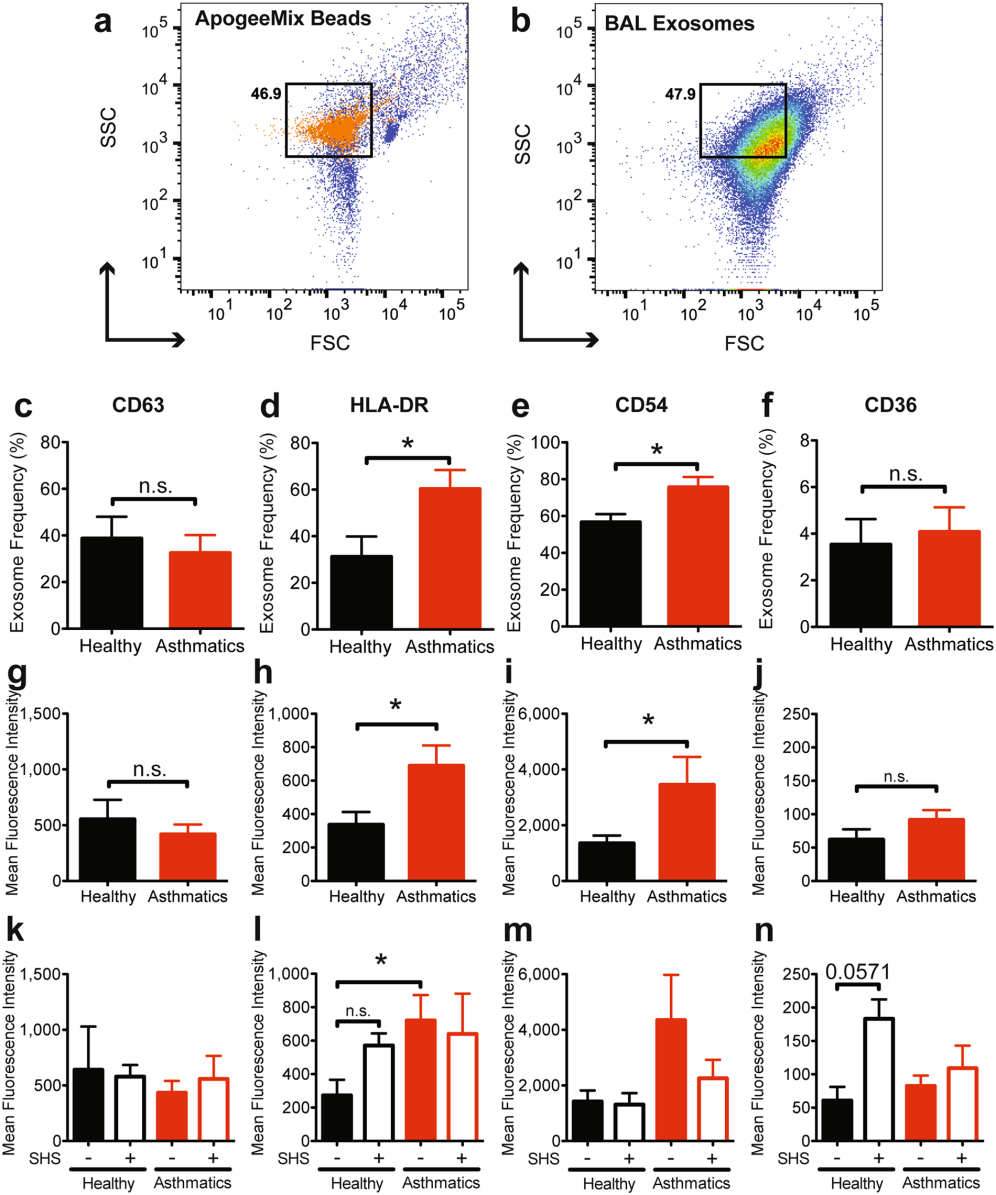
Characterization of EVs by flow cytometry. (**a**) ApogeeMix beads used to calibrate a BD LSRII to identify location of exosomes based on size. (**b**) A representative forward and side scatter of BAL EVs. (**c**) Frequency of CD63^+^ EVs. Mann Whitney T test, * < 0.05. (**d**–**f**) Frequency of EVs positive for HLA-DR, CD54, or CD36, after gating on CD63^+^ EVs. Mann Whitney T test, * < 0.05. (**g**,**h**) Mean fluorescence intensities of each marker analyzed on BAL EVs. Black bars represent healthy subjects (including SHS-exposed), and red bars represent asthmatics (including SHS-exposed). Mann Whitney T test, * < 0.05. (**k**–**n**) Mean fluorescence intensities of each marker analyzed on BAL EVs for each study group. Solid black bars represent healthy subjects, open black bars represent healthy subjects exposed to SHS, solid red bars represent asthmatics, and open red bars represent asthmatics exposed to SHS. Mann Whitney T test, * < 0.05; (Healthy Subjects, n = 9; Asthmatic Subjects, n = 9).

Tetraspanins, adhesion molecules and antigen presentation molecules have been identified as surface markers for exosomes^76^. Specifically, CD63, CD54, CD36, and HLA-DR have previously shown to be expressed on exosomes from BAL fluid^50,52^. Flow cytometry analysis showed that the proportion of BAL EVs positive for CD63 and staining intensity (MFI) of this marker were not significantly different between asthmatics and healthy subjects (Fig. 2g,k and Supplementary Fig. 1). Asthmatics had significantly higher frequencies of HLA-DR^+^ EVs (Fig. 2d), as well as increased expression of HLA-DR (Fig. 2h,l and Supplementary Fig. 1). CD54, also known as ICAM-1, is an adhesion molecule important for cell-to-cell adhesion and immune cell interactions. Frequencies of CD54^+^ EVs (Fig. 2e), and MFI (Fig. 2i and Supplementary Fig. 1) were also significantly higher in asthmatics as compared to healthy controls. CD36 is a pattern-recognition scavenger protein known to bind to phospholipids, lipoproteins, and oxidized lipids^77^. Differences observed between asthmatics and healthy subjects for CD36^+^ EVs (Fig. 2f) and MFI (Fig. 2j) were not statistically significant, however, SHS-exposed healthy patients had higher MFI (Fig. 2n). We further confirmed expression of these markers on EVs using ImageStream (Supplementary Fig. 2) confirming our observations from flow cytometry.

### Heterogeneity of Airway EVs

As EVs include both microvesicles and exosomes, we determined the quality and heterogeneity of our BAL EVs using flow cytometry. We used additional markers CD81, CD9, and TSG101 to mark exosomes. ARF6 is a plasma membrane protein present on microvesicles and therefore can help differentiate microvesicles from exosomes which are endosomally-derived^78^. Additionally, GRP94, a marker of endoplasmic reticulum was included to determine the quality of our EV preparations. GRP94^+^ EVs would suggest contamination by burst cells during EV isolation^79^.

**Figure 3:**
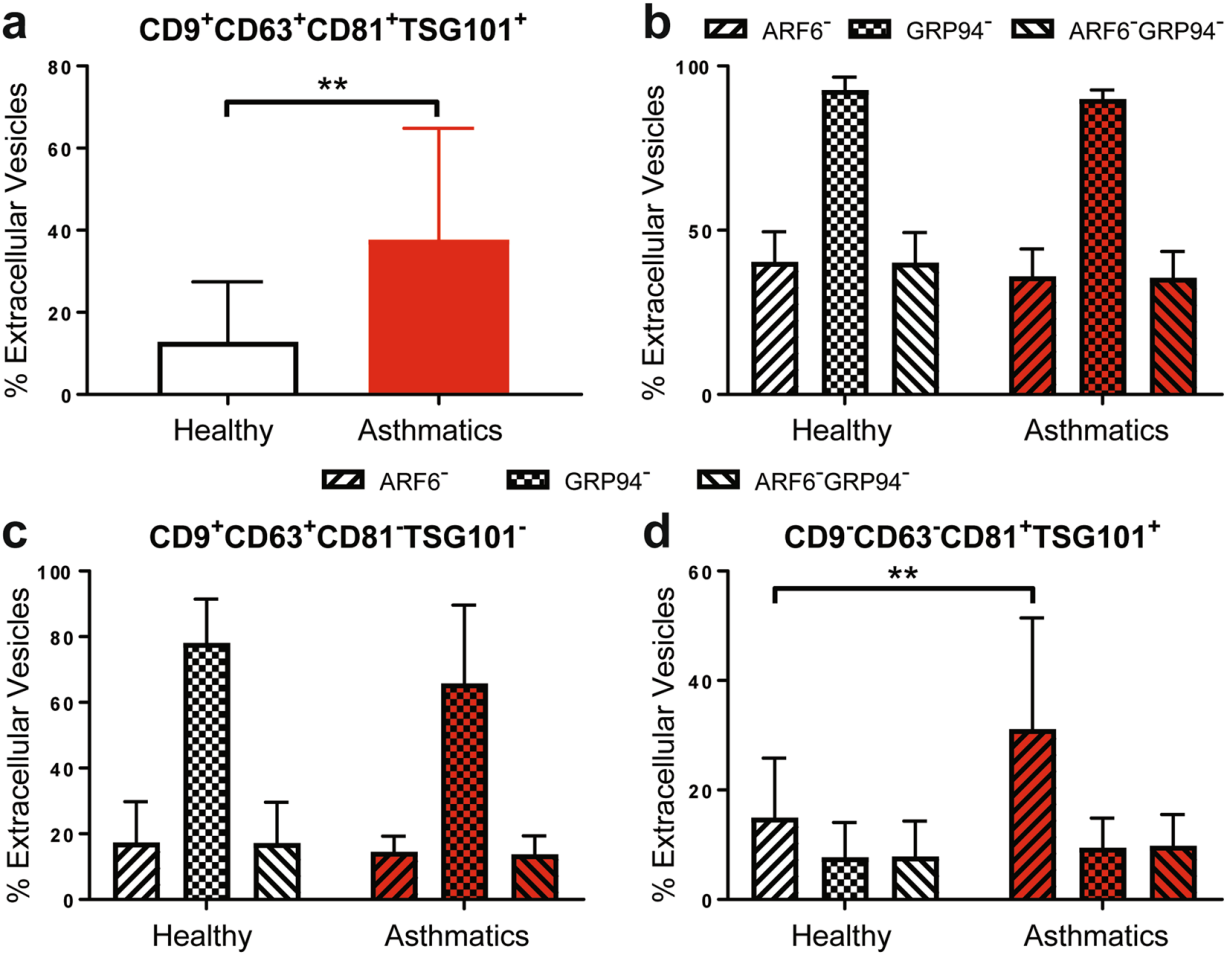
Heterogeneity of isolated BAL EVs. (**a**) The proportions of EVs which are CD9^+^CD63^+^CD81^+^TSG101^+^. Mann Whitney T test, ** < 0.01. (**b**) The proportions of ARF6^−^, GRP94^−^, and ARF6^−^GRP94^−^ EVs. (**c**) The proportions of ARF6^−^, GRP94^−^, and ARF6^−^GRP94^−^ EVs that are CD9^+^CD63^+^CD81^−^TSG101^−^. (**d**) The proportions of ARF6^−^, GRP94^−^, and ARF6^−^GRP94^−^ EVs that are CD9^−^CD63^−^CD81^+^TSG101^+^. Mann Whitney T test, ** < 0.01. (Healthy Subjects, n = 8; Asthmatic Subjects, n = 7).

In our airway EVs, the total percentage of EVs that are CD9^+^CD63^+^CD81^+^TSG101^+^ were significantly increased in asthmatics (Fig. 3a). We then determined percentages of ARF6^−^, GRP94^−^, and ARF6^−^GRP94^−^ airway EVs (Fig. 3b). Nearly 100% of EVs were GRP94^neg^ indicating that EVs did not have contaminating organelles from lysed cells. Furthermore, the proportion of both ARF6^−^ and ARF6^−^GRP94^−^ airway EVs were about the same, constituting 40% of the isolated EVs, thereby suggesting 40% of the airway EVs are exosomes.

We then assessed the proportion of ARF6^−^, GRP94^−^, and ARF6^−^GRP94^−^ airway EVs that are either CD9^+^CD63^+^CD81^−^TSG101^−^ or CD9^−^CD63^−^CD81^+^TSG101^+^ (Fig. 3c,d) which will comprehensively include all exosomes. We chose these two, combinatorial subset of exosome markers as a subset of study subjects had either CD9^+^CD63^+^ or CD81^+^TSG101^+^ EVs but not both. No clinical correlation or relationship with environmental factors, such as SHS exposure, could be associated with these study subjects. Approximately 20% of airway EVs were CD9^+^CD63^+^ and both ARF6^−^ and ARF6^−^GRP94^−^. Interestingly, within the CD9^−^CD63^−^CD81^+^TSG101^+^ airway EVs, 10% were ARF6^−^GRP94^−^ and not different between the study groups, while CD9^−^CD63^−^CD81^+^TSG101^+^ airway EVs that were just ARF6^−^ were significantly higher in asthmatics compared to healthy controls. Together, these results indicate that a heterogenous population of exosomes and microvesicles constitute the BAL EVs isolated from both healthy and asthmatic study subjects. Furthermore, there is a heterogeneous distribution of exosomes markers on the EVs that subset the exosomes within the BAL EVs.

### Airway EVs are enriched with lipids

As EVs are membrane bound particles, we determined qualitatively the relative abundance of lipids and proteins in airway EVs isolated from healthy and asthmatic subjects. Airway EVs were stained with PKH26, a lipophilic dye, and CFSE, which labels proteins. Both, airway EVs from healthy and asthmatic subjects, displayed higher MFI for PKH26 as compared to CFSE, suggesting that the EVs had relatively higher lipid content than proteins (Supplementary Fig. 3). No significant differences were observed in the relative quantity of lipids versus proteins between healthy and asthmatic subjects.

### The abundance of sphingomyelins, glycerophospholipids and ceramides in BAL EVs were significantly different between asthmatics and healthy subjects

Lipid mediators are involved in the inflammatory responses associated with asthma^26,27,34^. To evaluate whether airway EVs contain novel functional lipid mediators that may drive inflammatory responses in asthma, we performed an untargeted lipidomics analysis to characterize the lipid composition of airway EVs. We identified 529 lipids from the initial SWATH analysis (Supplementary Table 1). This complete list of lipids was then stratified based on significance to yield a list of 91 lipids (Supplementary Table 2). The initial bivariate analysis revealed 10 significantly different lipids (Table 2, Supplementary Fig. 4).

**Table 2:** Lipids with significant difference in abundance between study groups.

Lipid	Lipid ID	A (N = 6)		AS (N = 5)		H (N = 4)		HS (N = 5)		Wilcoxon Rank Test (one-tail)			
		Mean	Median	Mean	Median	Mean	Median	Mean	Median	A v H	H v HS	A v AS	HS v AS
Sphingomyelin 34:1	**LIPID162**	0.328	−0.620	0.086	−0.170	0.009	−0.224	−0.487	−0.623	0.415	0.204	0.327	**0.046**
Phosphatidylglycerol 34:2	***LIPID244***	−0.586	−0.872	−0.065	0.174	1.470	1.661	−0.407	−0.872	**0.023**	**0.040**	0.104	0.232
Monosialoganglioside 28:3	***LIPID58***	0.475	0.593	−0.591	−0.547	0.909	0.889	−0.706	−0.681	0.303	**0.024**	0.126	0.271
Monosialoganglioside 28:3	***LIPID59***	0.425	0.400	−0.613	−0.590	0.940	0.902	−0.648	−0.645	0.303	**0.024**	0.126	0.500
Ceramide-phosphate 28:1	***LIPID61***	0.584	0.629	−0.662	−0.663	0.930	0.940	−0.783	−0.756	0.237	**0.024**	0.055	0.341
Ceramide-phosphate 28:1	***LIPID63***	0.552	0.612	−0.648	−0.660	0.890	0.969	−0.728	−0.679	0.237	**0.024**	0.055	0.419
Mannosyl-diinositol phosphoryl-ceramide 26:2	**LIPID82**	0.528	0.494	−0.689	−0.696	0.927	1.064	−0.687	−0.704	0.378	**0.024**	0.074	0.500
Mannosyl-diinositol phosphoryl-ceramide 26:2	**LIPID83**	0.512	0.408	−0.668	−0.672	0.878	0.976	−0.649	−0.673	0.237	**0.024**	0.074	0.500
Ceramide 34:2	***LIPID86***	0.840	0.415	−0.714	−0.607	0.681	0.721	−0.840	−1.184	0.378	**0.023**	**0.012**	0.417
Ceramide-phosphate 28:0	***LIPID88***	0.568	0.613	−0.717	−0.709	0.974	1.005	−0.744	−0.740	0.378	**0.024**	**0.042**	0.420

Bivariate analyses determined statistically significant differences in abundance of lipids. A, asthmatic; AS, asthmatic exposed to SHS; H, healthy subject; HS, healthy subject exposed to SHS.

**Figure 4:**
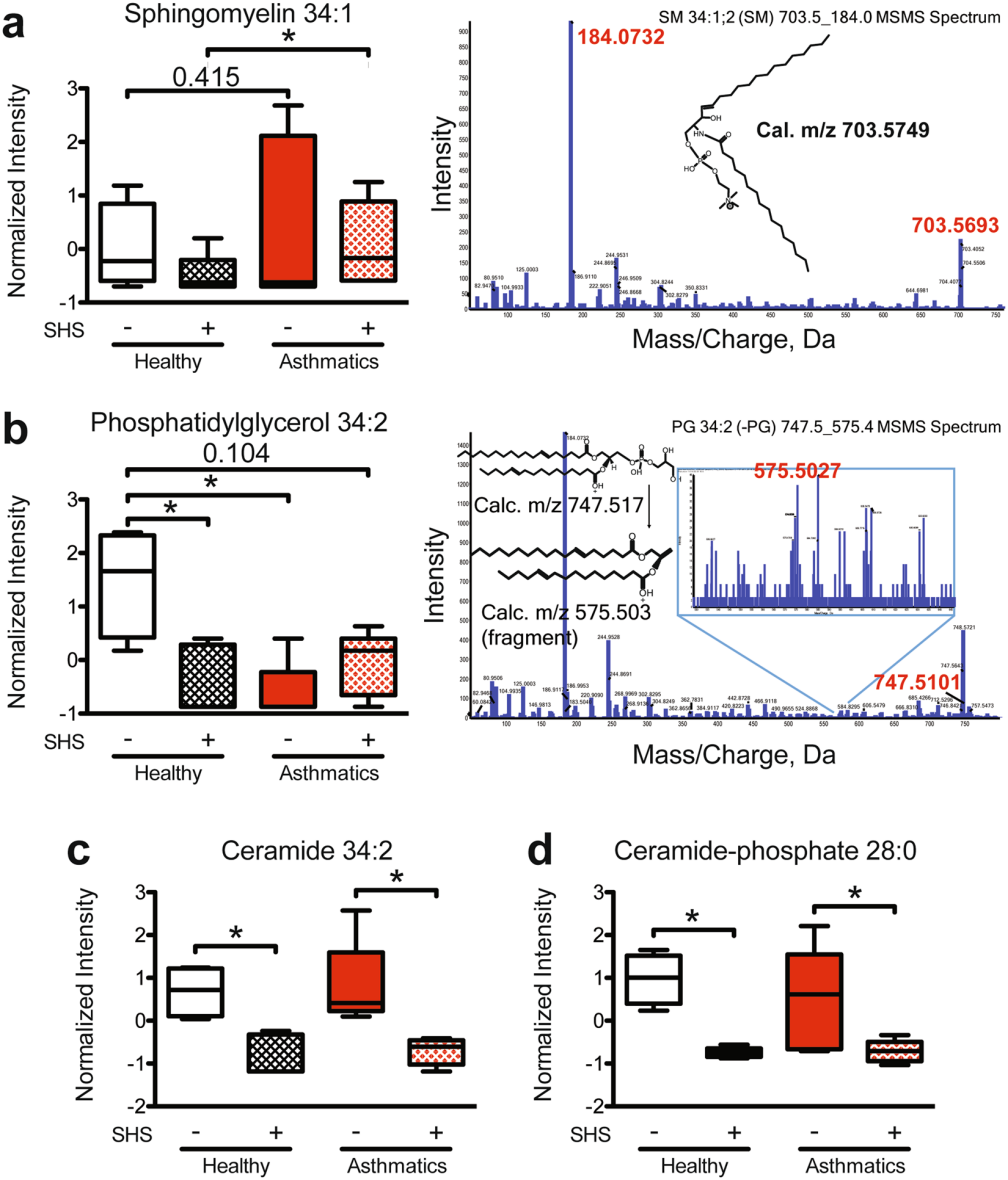
Significant differences in phosphoglycerolipids and sphingolipids in BAL EVs comparing study groups. (**a**) The intensity of abundance of sphingomyelin 34:1 in healthy SHS-exposed subjects compared to asthmatics exposed to SHS. Mann Whitney T test, * < 0.05. Corresponding mass spectrum and proposed structure shown to the right. (**b**) The intensity of abundance of phosphatidylglycerol 34:2 in SHS-exposed healthy subjects and non-SHS-exposed asthmatics as compared to non-SHS-exposed healthy subjects. Mann Whitney T test, * < 0.05. Corresponding mass spectrum and proposed structure shown to the right. (**c**) The intensity of abundance of ceramide 34:2 in SHS-exposed and non-exposed study groups. Mann Whitney T test, * < 0.05. (**d**) The intensity of abundance of ceramide-phosphate 28:0 in SHS-exposed and non-exposed study groups. Mann Whitney T test, * < 0.05; (Healthy Subjects, n = 9 (n = 4, no SHS; n = 5, + SHS); Asthmatic Subjects, n = 11 (n = 6, no SHS; n = 5, + SHS)).

Sphingomyelin, an important component of the plasma membranes^80^ and lipid rafts^81^, was significantly increased in the BAL EVs from asthmatics exposed to SHS, compared to SHS-exposed healthy subjects. Additionally, the same trend was observed in non-exposed asthmatics compared to healthy subjects with no SHS-exposure (Fig. 4a). Interestingly, phosphatidylglycerols, a known component of pulmonary surfactant^82^, were significantly lower in BAL EVs from non-exposed asthmatics compared to non-exposed healthy controls. The same lipid was significantly different in comparisons between SHS-exposed healthy subjects to and non-exposed controls (Fig. 4b). Lastly, ceramide 34:2, and ceramide-phosphate 28:0 were significantly reduced in BAL EVs from SHS-exposed groups as compared to their non-exposed counterparts (Fig. 4c and f). SWATH analyses were further validated by targeted analyses and confirmation of mass spectrometry peaks and structures were predicted for these lipids as shown.

**Figure 5:**
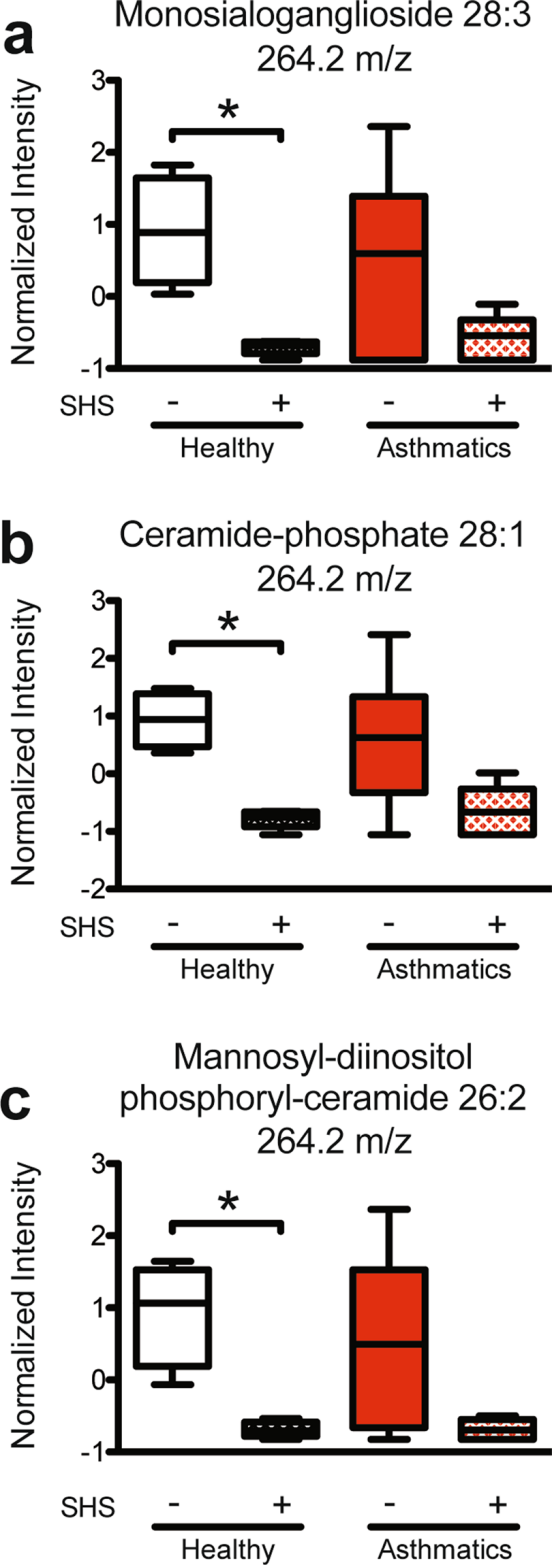
Ceramides were significantly different between healthy subjects and SHS-exposed healthy subjects. (**a**) Intensity of abundance of monosialoganglioside 28:3. Mann Whitney T test, * < 0.05. (**b**) Intensity of abundance of ceramide-phosphate 28:1. Mann Whitney T test, * < 0.05. (**c**) Intensity of abundance of mannosyl-diinositol phosphoryl-ceramide 26:2. Mann Whitney T test, * < 0.05; (Healthy Subjects, n = 9 (n = 4, no SHS; n = 5, + SHS); Asthmatic Subjects, n = 11 (n = 6, no SHS; n = 5, + SHS)).

Lipids that were significantly different between BAL EVs from SHS-exposed vs. non-exposed healthy subjects are listed in Table 1. In particular, monosialoganglioside 28:3, ceramide-phosphate 28:1, and mannosyl-diinositol phosphoryl-ceramide 26:2 were all significantly reduced only in the BAL EVs from healthy SHS-exposed subjects, as compared to the healthy non-SHS-exposed subjects (Fig. 5a–c). Additionally, the same lipid species were identified with different mass fragments differing by a water molecule (data not shown). These lipids also had the same trends and statistical significances. Ceramides are well characterized signaling molecules and regulators of inflammation^28,83^ and are also implicated in biogenesis of exosomes^66^. In our studies, we noted that ceramide-phosphate 28:1 was modified. We further validated our SWATH analysis by performing a targeted analysis for ceramide-phosphate 28:1, confirming its detection (Supplementary Fig. 5). Additionally, trihexosylceramide 40:1 was differentially detected between SHS-exposed and non-exposed healthy subjects. Trihexosylceramides have been reported to worsen COPD exacerbations^84^, and correlates with inflammation in obese diabetics^85^, although much is yet to be known about this lipid species. We also performed targeted lipidomics to validate our SWATH findings (Supplementary Fig. 6).

**Figure 6:**
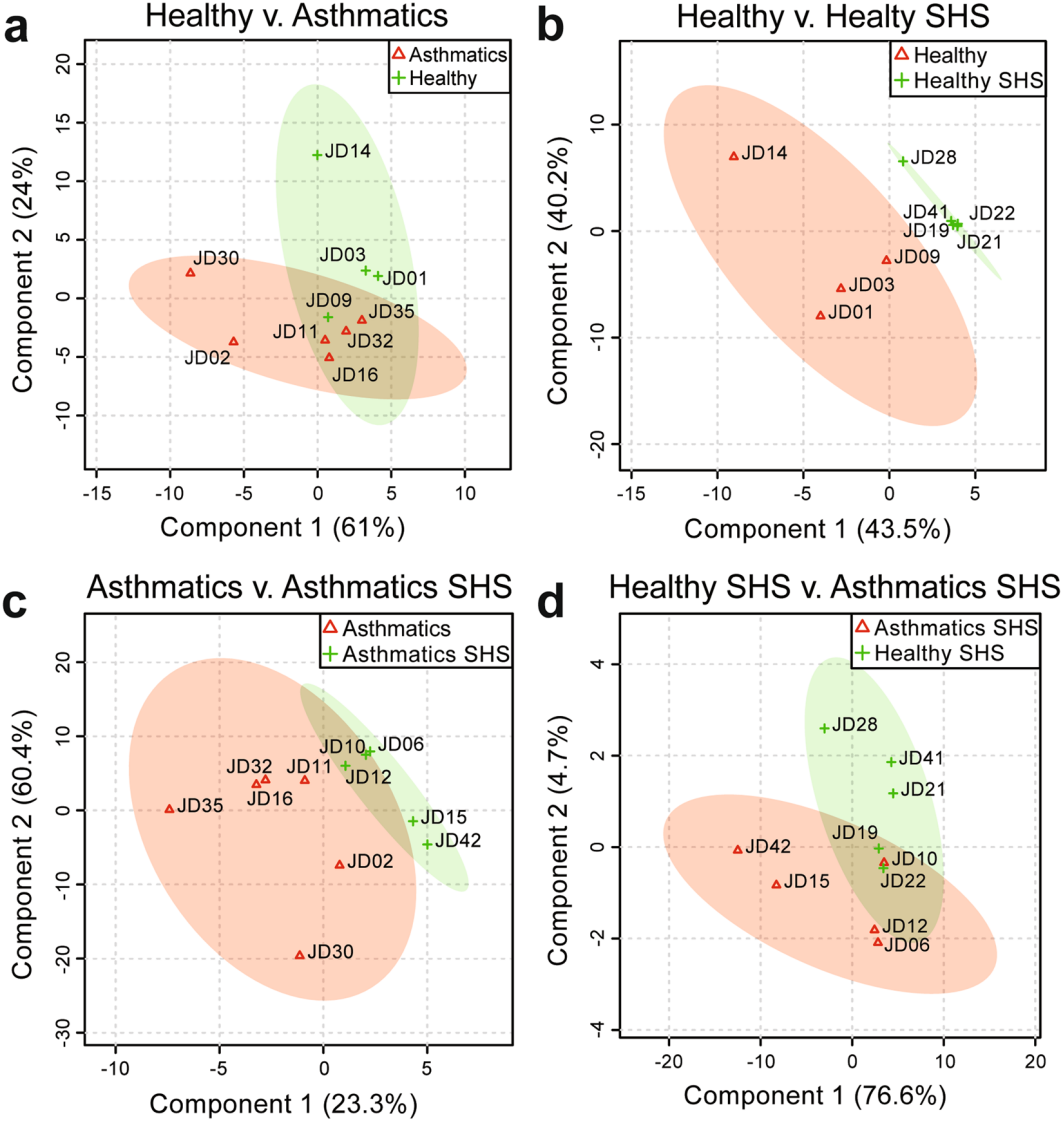
Partial least squares (PLS) discriminant analysis of lipids extracted from EVs. (**a**) Comparison of lipids identified in asthmatics and healthy subjects. (**b**) Comparison of lipids identified in healthy subjects and healthy SHS-exposed subjects. (**c**) Comparison of lipids identified in asthmatics and asthmatics exposed to SHS. (**d**) Comparison of lipids identified in asthmatics exposed to SHS and healthy subjects exposed to SHS, (Healthy Subjects, n = 9 (n = 4, no SHS; n = 5, + SHS); Asthmatic Subjects, n = 11 (n = 6, no SHS; n = 5, + SHS)).

Partial least squares (PLS) discriminant analysis confirmed that the 10 significantly different lipids were sufficient to separate each of our study groups. Phosphatidylglycerol alone was sufficient to separate asthmatics from healthy subjects in our study population (Fig. 6a and Supplementary Fig. 7a). Multivariate analysis also confirmed that ceramide-phosphate 28:1 and ceramide-phosphate 28:0 contributed significantly to the separation of healthy subjects from healthy subjects exposed to SHS (Fig. 5b and Supplementary Fig. 7b). Ceramide-phosphate 28:0 was also significant in the multivariate analysis between asthmatic subjects and asthmatics exposed to SHS (Fig. 6c and Supplementary Fig. 7c). In addition to ceramide-phosphate 28:0, ceramide 34:2 was very important in separating asthmatic subjects from asthmatics exposed to SHS (Fig. 6c and Supplementary Fig. 7c). Lastly, while the bivariate analysis revealed sphingomyelin 34:1 as significantly different between the groups, the PLS discriminant analysis showed two other sphingomyelins as important players in delineating the study groups, specifically sphingomyelins 42:3 and sphingomyelin 32:1 (Fig. 6d and Supplementary Fig. 7d). These results thus provide new insights into the altered lipid composition of airway EVs due to disease or SHS exposure.

## Discussion

Our study has identified characteristic differences of EVs from healthy subjects and asthmatics. We report, for the first time, significant differences in the lipid composition of EVs between heathy subjects, SHS-exposed healthy subjects, asthmatics, and SHS- exposed asthmatics. Lipids whose abundances were altered based on pathology or SHS-exposure included ceramides, ceramide-phosphates, phosphatidylglycerol, and sphingomyelins. The abundance of these lipids was not only significantly different between groups, as determined by bivariate analysis, but they also helped discriminate study groups. These results, combined with reports of these lipids as mediators of inflammation in asthma^20,26,86–88^, implicate a putative, albeit significant, role for EVs as a mode for transferring biologically active lipids.

Our observation that asthmatics have higher concentration of EVs in BAL fluid than healthy subjects aligns with previous reports^52^. The increased release of EVs may either be accounted for by increased production of EVs by airway cells of asthmatics, or increased immune cell infiltrates in the airways of asthmatics. Immune cell infiltration and increased eosinophilia are well documented in the airways of asthmatics^89^. We show that eosinophilia correlates with EV concentrations, and that both expression and frequencies of HLA-DR^+^ EVs were significantly increased in asthmatics. Both these observations support the enhancement of EVs in asthmatics.

In addition to the increased EV concentration, we report an increased frequency of HLA-DR^+^ EVs as well as HLA-DR expression on EVs from asthmatics consistent with published reports on BAL exosomes characteristics^50,52^. These data also suggest the increased potential for antigen presentation by EVs from asthmatics. Classically, HLA-DR is a class II molecule on the surface of antigen presenting cells that present antigens to T helper cells^90^. However more recently, it is being appreciated that class II presentation molecules can be present on EVs and transferred to other cells^91,92^, or directly stimulate T cells^93^. Direct activation of CD8^+^ T cells has been reported by exosomes purified from peptide pulsed monocyte-derived dendritic-cells^93^. Thus, the potential for airway EVs to promote T cell-mediated inflammation by direct stimulation is plausible.

CD63 is a tetraspanin molecule and an important scaffold protein known to play a role in structuring the immunological synapse, which is critical for effective immune signaling^94^. No significant differences were observed between asthmatics and health individuals in our study. CD36 is a receptor previously described to be associated with tocopherol signaling and lipid uptake^95^. Although we did not see significant changes in expression between healthy and asthmatic subjects, others have reported increased expression of both CD36 and CD63 on exosomes from asthmatic subjects^52^. Our patient population included a significant number of SHS-exposed individuals which may influence the differences in our observations.

From flow cytometry analyses, we also observe significantly higher frequencies of CD54^+^ EVs and increased MFI of CD54 on BAL EVs from asthmatics. CD54 has been shown to be increased in the airway of asthmatics potentially playing a role in asthma pathogenesis^96^. This data also parallels with reports that CD54 expression is increased on the airway epithelial cells of asthmatics^68^. The increased CD54^+^ BAL EVs in asthmatics may aid in EV-cell adhesion and signaling, and consequently immune activation^97^. Additionally, HLA-DR^+^CD54^+^ EVs may be derived from airway epithelial cells that have undergone spontaneous HLA-DR expression^68^, and increased CD54 expression due to inflammatory mediators, such as TNF-α and IL-4^98^. Further studies with additional characterization of BAL EVs to identify potential cellular origins may help us gain insights into the complex signaling and cytokine networks involved in asthma pathogenesis.

Additionally, we characterized for the first time the heterogeneity of airway EVs. Flow cytometry analysis with microvesicle marker ARF6 and ER marker GRP94, as well as other endosomally enriched markers (CD9, TSG101, CD81, and CD63) revealed that approximately 40% of the EVs purified from BAL are exosomes. Furthermore, the proportion of TSG101^+^CD81^+^ EVs were significantly increased in asthmatics suggesting that certain exosome markers may be specifically enriched in EVs related to pathology. More importantly, the use of GRP94 in our analysis has confirmed the quality of the EV isolation, indicating very minimal contamination from the endoplasmic reticulum of lysed cells.

We next sought out to measure the relative abundance of lipids and proteins in airway EVs using PKH26, a lipophilic dye, and CFSE, which stains proteins. The CellTrace CFSE requires intracellular esterase to remove the acetate group, generating a fluorescent ester. Because we were able to acquire signal for CFSE using ImageStream, this indirectly suggests that there are functional esterase enzymes contained in the airway EVs from our subjects. Interestingly, we observed that lipids were in relatively high abundance as compared to proteins in both airway EVs from asthmatics and healthy subjects. This led us to investigate if EVs are curriers of bioactive lipids, warranting a lipidomics analysis to better characterize airway EVs.

Our SWATH lipidomics analysis of the BAL EVs revealed unique differences in the abundance of lipids found in airway EVs between study groups. Recent studies have shown the involvement of certain classes of lipids in pulmonary inflammation and disease^99,100^. For example, lack of balance between ceramides and sphingomyelin, such as the one caused by ORMDL3 polymorphism in humans, can promote airway hyper-reactivity by increasing ceramide levels in the lung^101^. Two major classes of lipids were identified to be significantly different in our studies: sphingolipids and phosphoglycerolipids. Phosphoglycerolipids are known components of airway surfactants^82^, while sphingolipids are potent signaling molecules that play an important role in inflammation and immune response^102^. Interestingly, the abundance of phosphatidylglycerol 34:2 was significantly reduced in the SHS-exposed study groups, and in asthmatic patients. Altered surfactant composition is known to reduce lung function in asthma^103^, and this change that was detected in BAL EVs may indicate such alterations in surfactant content. Sphingolipids, such as ceramides, are important for lipid raft formation that are necessary for functional immune signaling^34,104^. Additionally, ceramides are critical for the formation of exosomes^66^, and inhibition of sphingomyelinases, which produce ceramides from sphingomyelins^105^, results in reduction of exosome formation^66^. Together, alterations in the lipid composition of EVs may not only affect the metabolism and cellular function of target cells, but also reflect the state of the parental cell and its packaging capabilities.

In addition to changes in phosphoglycerolipids, ceramide levels were reduced in BAL EVs from SHS-exposed asthmatics and healthy subjects. Interestingly, in animal models of cigarette smoke exposure or SHS-exposure, ceramide levels were higher in serum and BAL fluid^99,100^. Although these reports indicate an accumulation of ceramides in the serum and BAL fluid, the changes in EV lipid content observed in our study may represent a snapshot of the cellular metabolic flux for ceramides. Lastly, a study using both a murine model of cigarette smoke injury and *ex vivo* human cells, demonstrated that exogenous addition of ceramide-phosphate reduced lung inflammation induced by cigarette smoke^83^. Interestingly, we observed that ceramide-phosphates were reduced in BAL exosomes from both healthy and asthmatic subjects exposed to SHS. Thus, ceramide-phosphates may play an endogenous protective anti-inflammatory role, which is impaired due to SHS exposure. Although our lipidomics analyses only reports on relative abundance of the identified lipids, several studies have reported on concentrations of these identified lipids in airway diseases and lung injury including cystic fibrosis, acute respiratory distress syndrome and pneumonia^106–108^.

Taken together our studies have, for the first time, characterized the lipid signatures of BAL EVs from asthmatics and healthy subjects, as well as SHS-exposed groups. Furthermore, the altered abundances in lipids we observed in the BAL EVs may not only reflect the state of cellular lipid metabolism, as compared between healthy and diseased, but also give us insights into functional lipids that could potentially be stably transported between cells via EVs. Various lipids, such as ceramides, prostaglandins, and leukotrienes, have been implicated in promoting inflammation in asthma^9,12,14,20,21^. Leukotrienes have been shown to be transported by EVs to target cells, to promote an inflammatory response. Additionally, the lipid composition of the lymphocyte plasma membrane is critical for the formation of immunological synapses^109^. Specifically, sphingomyelins and ceramides play an important role in T cell function by regulating plasma membrane fluidity^110^. Thus, antigen presentation by EVs may not be the only mechanism of EV-mediated T cell activation, as the transfer of lipids such as sphingomyelins and ceramides by exosomes may alter the plasma membrane rigidity and thereby influence the formation of an immunological synapse. Lastly, SHS exposure changed the composition of lipids in EVs potentially promoting inflammation and abrogating the protective cellular mechanisms by altering cellular lipid signaling and metabolism.

In summary, BAL EVs contain lipids that have been known to play an important role in cellular signaling, survival, and immune activation^21,87,88,104,111,112^. Furthermore, specific lipids, such as ceramides, sphingosines, prostaglandins and leukotrienes, have been identified to drive inflammation in asthma^9,14,20,26,86,99^. Thus, our findings open a new path for understanding a putative and novel role for EVs as reservoirs or intercellular transporters of pro-inflammatory lipids in asthma, thus identifying potentially new therapeutic targets.

## Electronic supplementary material

Supplementary Information

Supplementary Dataset
