# Supplementary material for: Unique Lipid Signatures of Extracellular Vesicles from the Airways of Asthmatics
Source: Sci Rep. 2018 Jul 9;8:10340. doi: 10.1038/s41598-018-28655-9 (PMC6037776; doi:10.1038/s41598-018-28655-9)
Supplement: Supplementary file 1 — Supplementary Information [file 41598_2018_28655_MOESM1_ESM.pdf]

Unique Lipid Signatures of Extracellular Vesicles from the Airways of Asthmatics

Hough, K.P., BS[1], L. Wilson, BS[4,5], J.L. Trevor, MD[1], J.G. Strenkowski, BS[1], N. Maina, MD, PhD[1], Y. Kim, PhD[1], M.L. Spell, BS[2], Y. Wang, PhD[1], D. Chanda, PhD[1], J. Rodriguez Dager BS[1], N. Sharma, MD[1], C. Miranda, MD, PhD[1], V.B. Antony, MD[1], M.T. Dransfield, MD[1], D.D. Chaplin, MD, PhD[3], C. Steele, PhD[1], S. Barnes, PhD[4, 5], S.R. Duncan, MD[1], J.K. Prasain, PhD[4, 5], V.J. Thannickal, MD[1], and J.S. Deshane, PhD[1]*

[1]Department of Medicine, Division of Pulmonary, Allergy and Critical Care Medicine, [2]Center for Aids Research, [3]Department of Microbiology, [4]Department of Pharmacology and Toxicology, and [5]Targeted Metabolomics and Proteomics Laboratory, University of Alabama at Birmingham, Birmingham, AL

Supplementary Figure 1

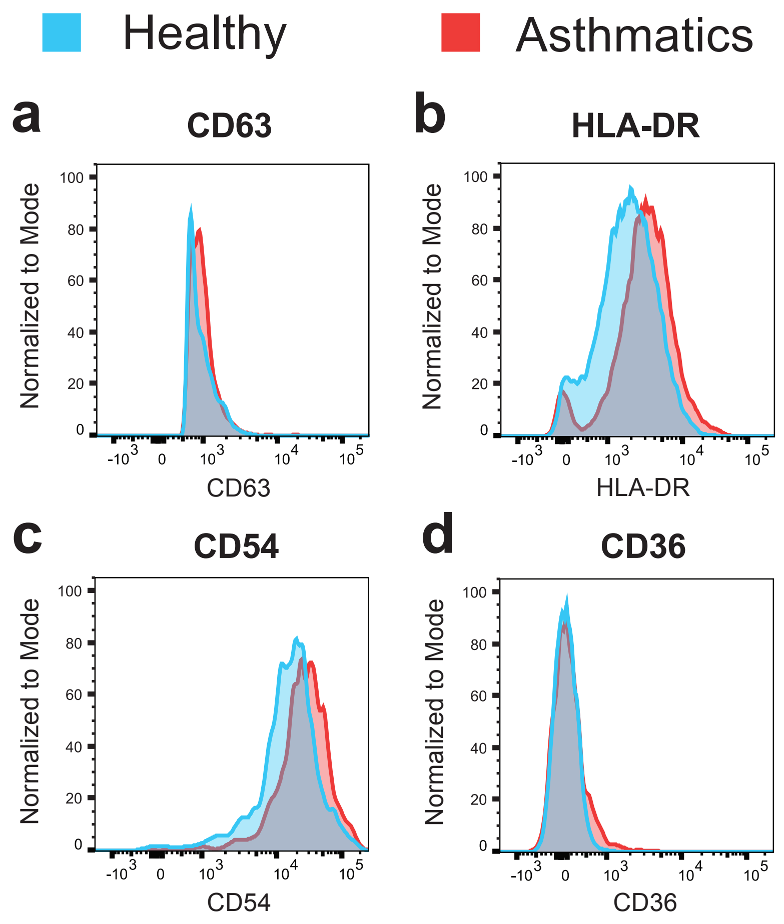

Supplementary Figure 1 – Representative histograms of airway exosomes stained with antibodies for surface markers: CD63, HLA-DR, CD54, and CD36. (a) CD63, a tetraspanin used as an exosome marker. (b) HLA-DR, a class II antigen presentation molecule. (c) CD54, also known as ICAM-1, a surface integrin molecule. (d) CD36, a pattern-recognition scavenger protein (Healthy subjects n=9; Asthmatic subjects n=9).

Supplementary Figure 2

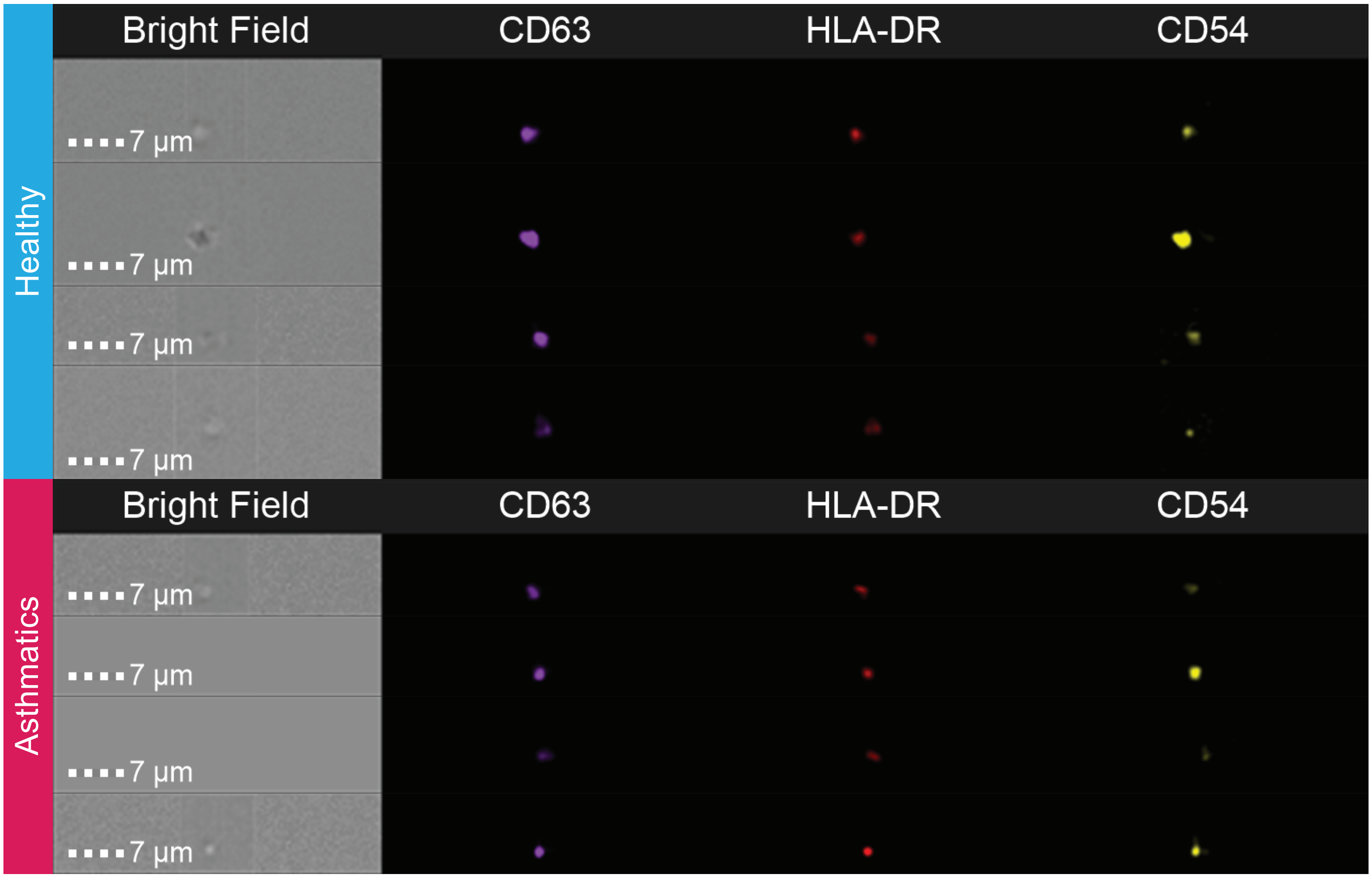

Supplementary Figure 2 – ImageStream analysis of airway exosomes from healthy and asthmatic subjects. (a) Representative fluorescence images from ImageStream of airway exosomes from four healthy and asthmatic subjects. Exosomes were stained with antibodies for surface markers: CD63, HLA-DR, and CD54 (Healthy subjects n=6; Asthmatic subjects n=6).

Supplementary Figure 3

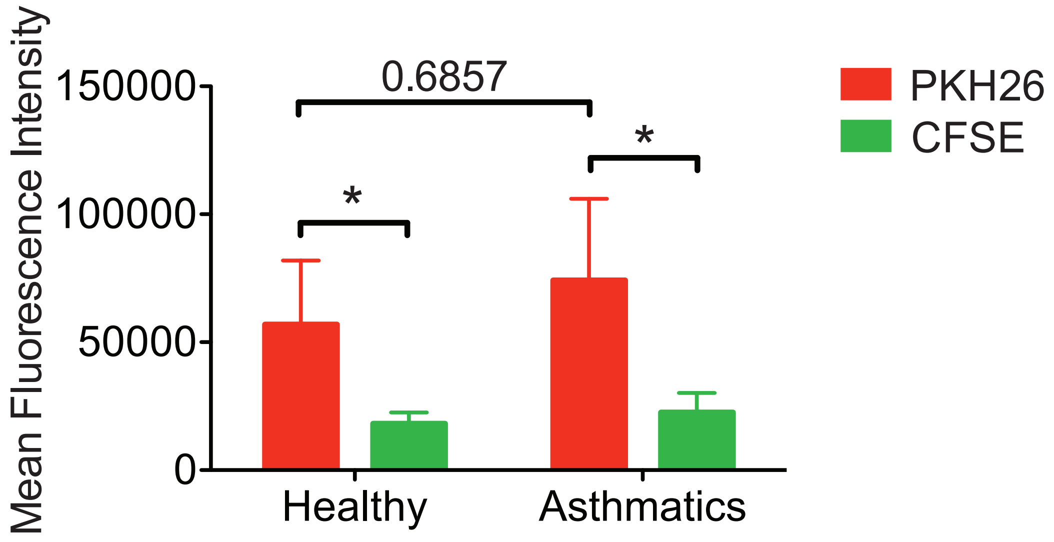

Supplementary Figure 3 – Airway exosomes have higher lipid content than protein, as assessed by PKH26, a lipophilic dye, and CFSE for protein using ImageStream. Differences in protein and lipid content was not seen between study groups (Mann Whitney T test, *<0.05; Healthy subjects n = 4; Asthmatic subjects n =4).

Supplementary Figure 4

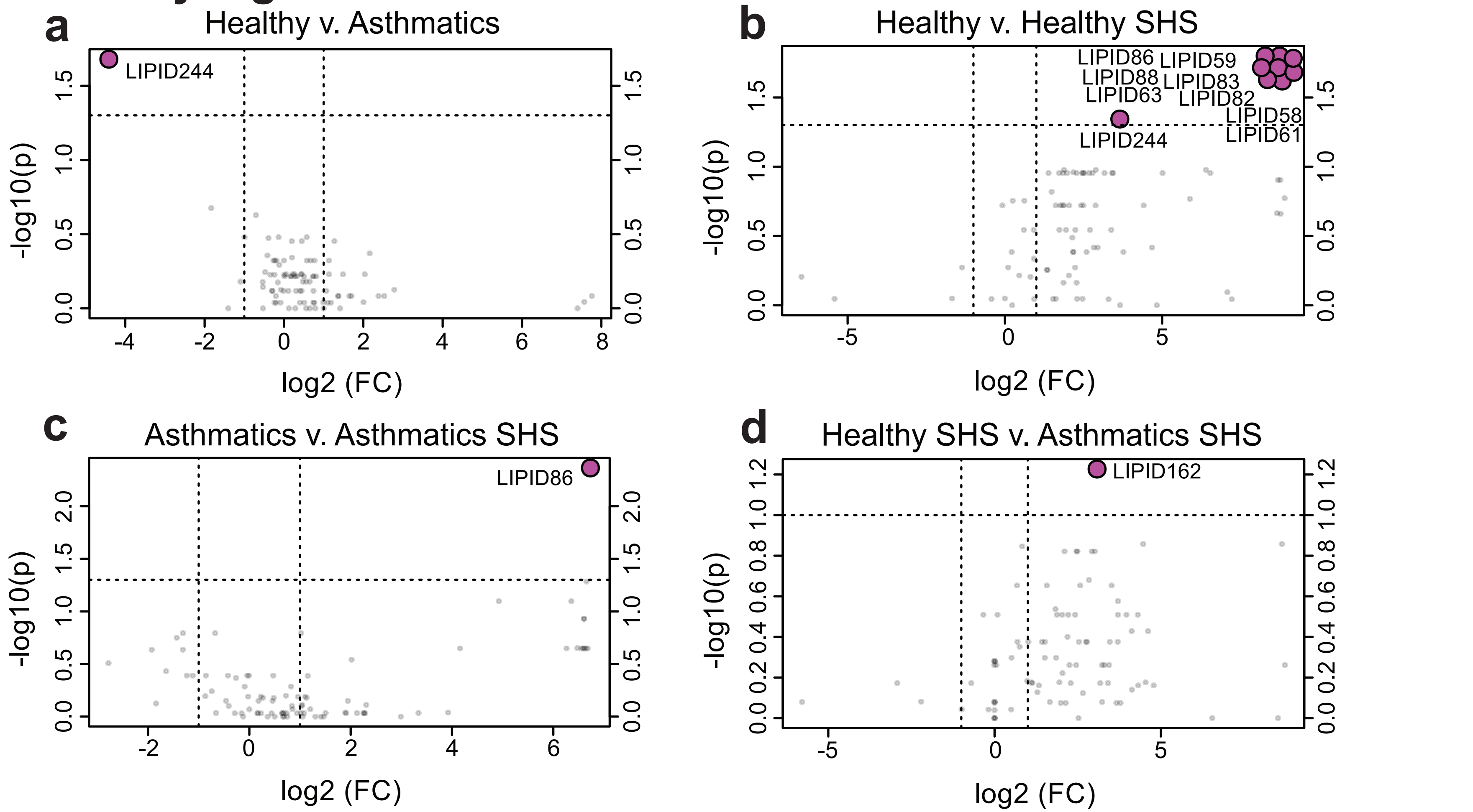

Supplementary Figure 4 – Volcano plot of the bivariate analysis (performed on Metaboanalyst 3.0) of identified lipids from BAL exosomes. Y-axis displays significance, represented as –log(p-value), and the x-axis displays log base-2 fold-change. The horizontal dotted line demarks the 0.05 p-value cutoff. Significant data points were enlarged and colored purple for illustration purposes. (a) Healthy subjects versus asthmatics, non-SHS exposed. LIPID244 (phosphatidylclycerol 34:2, p=0.023) was found to be more abundant in healthy subjects. (b) Healthy subjects versus SHS exposed healthy subjects. Identified lipids were all higher in healthy subjects. LIPID 58 (monosialoganglioside 28:3, 264.2 m/z, p=0.024), LIPID59 (monosialoganglioside 28:3, 282.2 m/z, p=0.024), LIPID61 (ceramide-phosphate 28:1, 264.2 m/z, p=0.024), LIPID63 (ceramide-phosphate 28:1, 282.2 m/z, p=0.024), LIPID82 (mannosyl-diinositol phosphoryl-ceramide 26:2, 264.2 m/z, p=0.024), LIPID83 (mannosyl-diinositol phosphoryl-ceramide 26:2, 282.2 m/z, p=0.024), LIPID86 (ceramide 34:2, p=0.023), LIPID88 (ceramide-phosphate 28:0, p=0.024), and LIPID244 (phosphatidylglycerol 34:2, p=0.040). (c) Asthmatics versus SHS exposed asthmatics. LIPID86 (ceramide 34:2, p=0.012) was significantly higher in asthmatics. (d) Healthy subjects exposed to SHS versus asthmatics exposed to SHS. LIPID162 (sphingomyelin 34:1, p=0.046) was significantly higher in asthmatics exposed to SHS (Healthy subjects n = 9 (no SHS n=4, +SHS n = 5); Asthmatic subjects n =11 (no SHS n=6, +SHS n = 5)).

Supplementary Figure 5

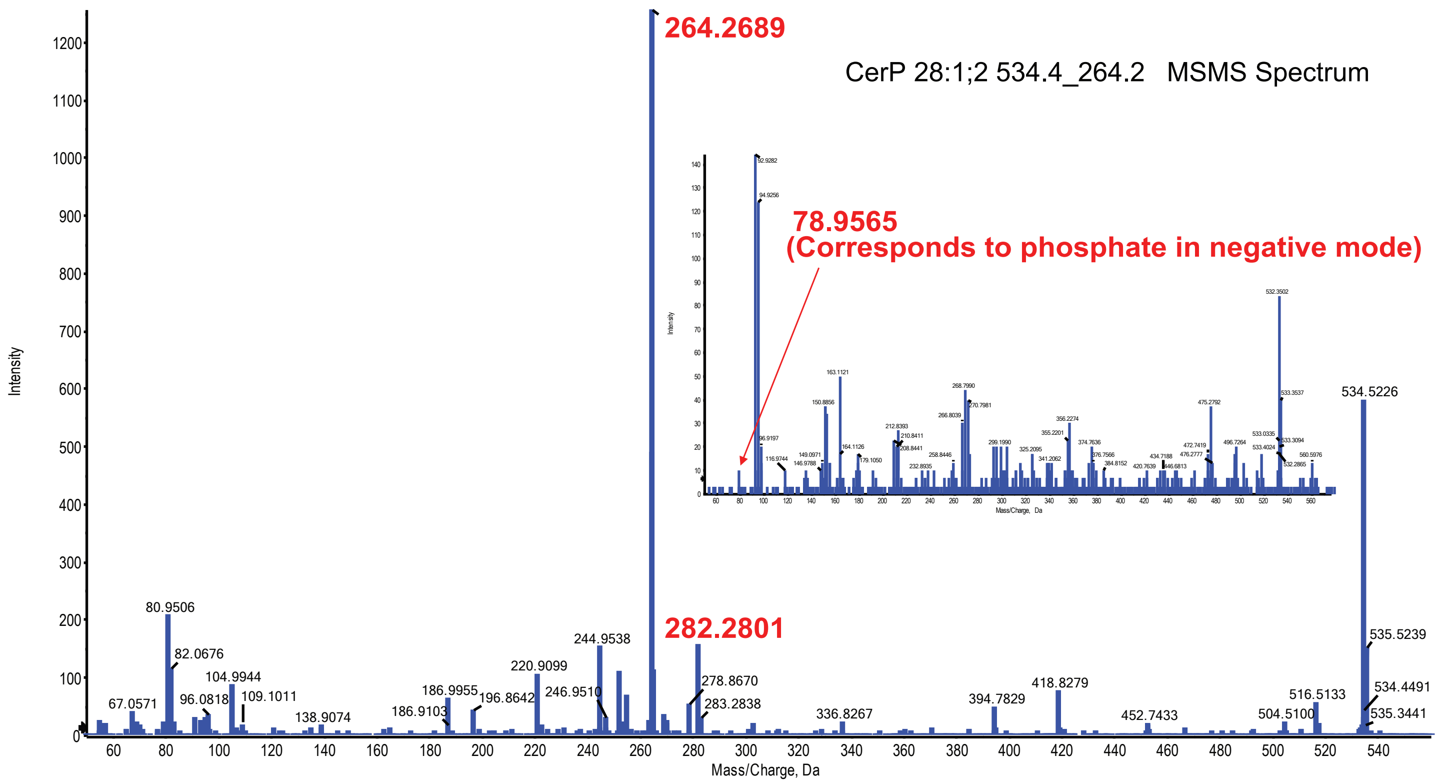

Supplementary Figure 5 – Mass spectrum of ceramide-phosphate 28:1 from BAL exosomes. Ceramide-phosphate 28:1 was detected from the SWATH lipidomics reported in Table 2. The precense of this modified lipid species was confirmed by targeted lipidomics. The red arrow pointing to the mass/charge ratio of 78.9565 m/z corresponds to the phosphate of ceramide-phosphate 28:1.

Supplementary Figure 6

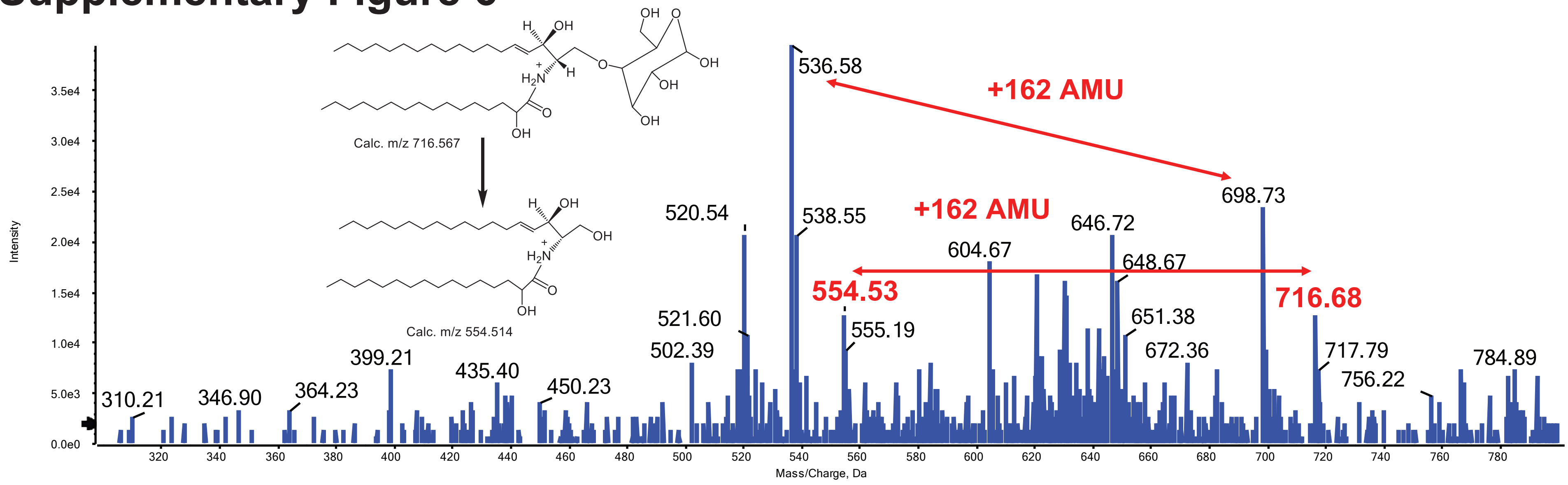

Supplementary Figure 6 – Mass spectrum of trihexosylceramide 40:1. Trihexosylceramide 40:1 was detected from the SWATH lipidomics reported in Supplementary Table 2. The precense of this lipid species was confirmed by targeted lipidomics. The proposed structure for trihexosylceramide 40:1 is also represented in the mass spectrum on the left-side. The red arrows indicate the mass difference for the hexose.

Supplementary Figure 7

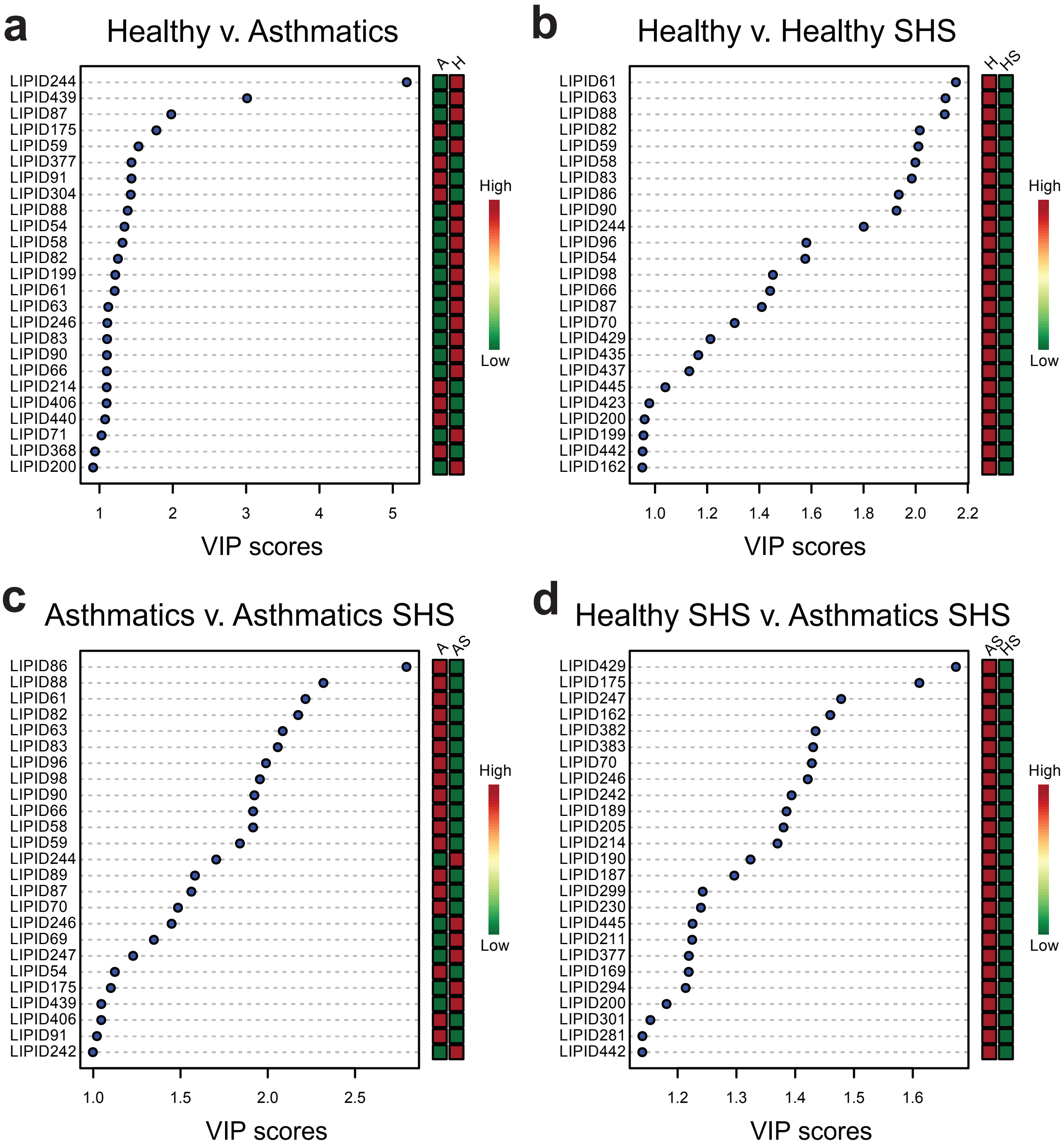

Supplementary Figure 7 – Variable Importance in Projection (VIP) scores from the partial least squares (PLS) analysis (performed on Metaboanalyst 3.0) of identified lipids from BAL exosomes. Higher VIP scores indicate important players in the PLS model. (a) Healthy subjects versus asthmatics, non-SHS exposed. The significant lipid, LIPID244, is phosphatidylglycerol 34:2. (b). Healthy subjects versus SHS exposed healthy subjects. The important lipids, LIPID61, LIPID62 and LIPID88, are ceramide-phosphate 28:1 (264.2 m/z), ceramide-phosphate 28:1 (282.2 m/z), and ceramide-phosphate 28:0, respectively. (c) Asthmatics versus SHS exposed asthmatics. The significant lipid, LIPID86, is ceramide 34:2, and LIPID88 ceramide-phosphate 28:0. (d) Healthy subjects exposed to SHS versus asthmatics exposed to SHS. The significant lipids, LIPID 429 and LIPID 175, are sphingomyelin 42:3 and sphingomyelin 32:1, respectively (Healthy subjects n = 9 (no SHS n=4, +SHS n = 5); Asthmatic subjects n =11 (no SHS n=6, +SHS n = 5)).

Supplementary Table 2 – List of 91 lipids and their bivariate analysis after filtering out study groups with missing values.

			A (N=6)		AS (N=5)		H (N=4)		HS (N=5)		Wilcoxon Rank Test				T-test			
Annotation	Expanded Annotation	LIPIDID	Mean	Median	Mean	Median	Mean	Median	Mean	Median	AS vs HS	A vs AS	A vs H	H vs. HS	AS vs HS	A vs AS	A vs H	H vs. HS
SM 34:1;2 (SM)	Sphingomyelin 34:1;2	LIPID162	0.328067	-0.62024	0.08551	-0.1695	0.009385	-0.2237	-0.4867	-0.62302	0.046	0.327	0.415	0.204	0.093	0.38	0.359	0.132
SM 34:0;2 (SM)	Sphingomyelin 34:0;2	LIPID169	0.199614	-0.52572	0.166211	-0.57729	0.010043	-0.49582	-0.41378	-0.5405	0.42	0.465	0.237	0.151	0.139	0.483	0.411	0.205
PC 30:0 (PC 104)	Phosphatidylcholine 30:0	LIPID172	0.130729	-0.4815	0.193291	-0.41277	-0.03944	-0.33733	-0.31861	-0.35339	0.341	0.429	0.458	0.452	0.196	0.468	0.417	0.295
PC 30:0 (LPC)	Lyso-Phosphatidylcholine 30:0	LIPID173	0.239603	-0.47404	0.172282	-0.53568	-0.08026	-0.46012	-0.3956	-0.56524	0.213	0.465	0.459	0.208	0.166	0.467	0.346	0.223
PC 30:0 (PC)	Phosphatidylcholine 30:0	LIPID174	0.202542	-0.52591	0.165802	-0.58867	-0.02696	-0.49692	-0.38728	-0.59127	0.213	0.465	0.303	0.208	0.171	0.481	0.389	0.236
SM 32:1;4 (SM)	Sphingomyelin 32:1;4	LIPID175	0.006023	-0.48047	0.733365	-0.23065	-0.44951	-0.4588	-0.38099	-0.48047	0.086	0.096	0.5	0.448	0.069	0.186	0.212	0.186
SM 34:1;3 (SM)	Sphingomyelin 34:1;3	LIPID187	-0.00639	-0.57357	0.394135	-0.54292	0.051572	-0.41876	-0.42773	-0.55456	0.343	0.213	0.301	0.075	0.123	0.303	0.467	0.174
PC O-32:0 (LPC)	Ether-linked Lyso-Phosphatidylcholine32:0	LIPID189	-0.01842	-0.55392	0.49132	-0.54714	-0.02947	-0.42859	-0.44564	-0.48321	0.5	0.213	0.301	0.453	0.106	0.261	0.493	0.161
PC O-32:0 (PC)	Ether-linked Phosphatidylcholine32:0	LIPID190	-0.04991	-0.56569	0.503803	-0.5428	-0.06804	-0.46602	-0.38948	-0.55483	0.213	0.213	0.301	0.151	0.118	0.242	0.489	0.237
PC 32:2 (LPC)	Lyso-Phosphatidylcholine 32:2	LIPID199	0.048595	-0.48451	-0.07785	-0.44018	0.541021	-0.27597	-0.41328	-0.56082	0.198	0.5	0.178	0.104	0.175	0.412	0.294	0.131
PC 32:2 (PC)	Phosphatidylcholine 32:2	LIPID200	0.104235	-0.49012	-0.08804	-0.42789	0.479676	-0.31669	-0.42078	-0.54783	0.198	0.39	0.133	0.072	0.147	0.377	0.343	0.129
SM 36:1;2 (SM)	Sphingomyelin 36:1;2	LIPID202	0.058751	-0.5535	0.253687	-0.61625	0.070411	-0.44239	-0.38052	-0.58693	0.273	0.395	0.378	0.151	0.17	0.396	0.494	0.231
PC 32:1 (PC 104)	Phosphatidylcholine 32:1	LIPID205	0.179646	-0.39343	0.004398	-0.32303	0.309763	-0.32218	-0.46778	-0.71241	0.062	0.329	0.339	0.072	0.107	0.399	0.443	0.135
PC 32:1 (LPC)	Lyso-Phosphatidylcholine 32:1	LIPID206	0.182795	-0.52196	0.007559	-0.53894	0.241813	-0.38985	-0.42036	-0.56927	0.213	0.465	0.237	0.075	0.166	0.403	0.474	0.16
PC 32:1 (PC)	Phosphatidylcholine 32:1	LIPID207	0.168765	-0.50335	0.03212	-0.56706	0.196362	-0.43476	-0.39173	-0.60688	0.162	0.5	0.303	0.075	0.185	0.424	0.487	0.19
SM 36:0;2 (SM)	Sphingomyelin 36:0;2	LIPID209	0.136552	-0.44011	0.167535	-0.6498	0.077198	-0.38203	-0.39316	-0.59264	0.42	0.5	0.459	0.107	0.191	0.484	0.469	0.211
PC 32:0 (PC 104)	Phosphatidylcholine 32:0	LIPID210	0.203123	-0.50813	0.071084	-0.55421	0.07285	-0.42544	-0.37311	-0.5225	0.5	0.465	0.303	0.075	0.186	0.433	0.44	0.204
PC 32:0 (LPC)	Lyso-Phosphatidylcholine 32:0	LIPID211	0.144765	-0.49553	0.23496	-0.59838	0.016965	-0.46349	-0.42225	-0.60805	0.273	0.465	0.378	0.151	0.138	0.453	0.436	0.207
PC 32:0 (PC)	Phosphatidylcholine 32:0	LIPID213	0.112204	-0.44366	0.239053	-0.65291	0.039017	-0.42797	-0.40491	-0.63818	0.273	0.465	0.378	0.107	0.16	0.434	0.462	0.215
SM 34:1;4 (SM)	Sphingomyelin 34:1;4	LIPID214	0.119147	-0.54443	0.444805	-0.5336	-0.22325	-0.52433	-0.40918	-0.55627	0.338	0.463	0.5	0.451	0.109	0.345	0.313	0.293
SM 36:2;3 (SM)	Sphingomyelin 36:2;3	LIPID230	0.100972	-0.52238	0.327811	-0.49884	-0.09289	-0.4885	-0.37466	-0.49331	0.341	0.358	0.5	0.451	0.135	0.389	0.401	0.265
PC O-34:1 (LPC)	Ether-linked Lyso-Phosphatidylcholine34:1	LIPID238	0.073018	-0.61687	0.230173	-0.53813	0.029717	-0.3886	-0.34157	-0.59145	0.162	0.267	0.301	0.279	0.173	0.418	0.478	0.265
PC O-34:1 (PC)	Ether-linked Phosphatidylcholine34:1	LIPID239	0.08819	-0.63942	0.19898	-0.57737	-0.00336	-0.46619	-0.30212	-0.58708	0.213	0.327	0.178	0.151	0.222	0.442	0.452	0.304
SM 36:1;3 (SM)	Sphingomyelin 36:1;3	LIPID242	-0.06249	-0.58553	0.573768	-0.47104	-0.11435	-0.48148	-0.4073	-0.58594	0.162	0.129	0.301	0.151	0.105	0.21	0.465	0.245
PG 34:2 (-PG)	Phosphatidylglycerol 34:2	LIPID244	-0.5864	-0.87154	-0.06524	0.174447	1.470416	1.661	-0.4074	-0.87154	0.232	0.104	0.023	0.04	0.202	0.076	0.001	0.006
PC O-34:0 (LPC)	Ether-linked Lyso-Phosphatidylcholine34:0	LIPID246	-0.24695	-0.5133	0.69478	-0.4699	-0.02253	-0.32544	-0.38042	-0.44731	0.5	0.167	0.335	0.5	0.1	0.116	0.311	0.191
PC O-34:0 (PC)	Ether-linked Phosphatidylcholine34:0	LIPID247	-0.13449	-0.5329	0.655011	-0.41714	-0.07827	-0.42038	-0.431	-0.48221	0.273	0.129	0.415	0.406	0.09	0.158	0.457	0.191
SM 38:3;2 (SM)	Sphingomyelin 38:3;2	LIPID262	0.300181	-0.45796	-0.11881	-0.44217	0.138948	-0.36181	-0.35257	-0.4346	0.341	0.358	0.335	0.204	0.175	0.296	0.433	0.177
PC 34:3 (LPC)	Lyso-Phosphatidylcholine 34:3	LIPID265	0.217167	-0.51735	-0.0427	-0.46471	0.197706	-0.38203	-0.37606	-0.4809	0.273	0.215	0.237	0.107	0.157	0.365	0.492	0.169
PC 34:3 (PC)	Phosphatidylcholine 34:3	LIPID267	0.191151	-0.46298	-0.17101	-0.45815	0.406196	-0.36141	-0.38332	-0.48526	0.162	0.464	0.181	0.075	0.178	0.29	0.411	0.145
SM 38:2;2 (SM)	Sphingomyelin 38:2;2	LIPID274	0.249917	-0.4908	-0.0188	-0.53867	0.149014	-0.44091	-0.40031	-0.55274	0.213	0.465	0.303	0.075	0.168	0.359	0.456	0.182
PC 34:2 (PC 104)	Phosphatidylcholine 34:2	LIPID280	0.232438	-0.48464	0.02925	-0.45786	-0.07021	-0.41976	-0.25201	-0.45786	0.377	0.266	0.459	0.453	0.272	0.401	0.368	0.352
PC 34:2 (LPC)	Lyso-Phosphatidylcholine 34:2	LIPID281	0.30286	-0.45456	-0.00251	-0.47557	0.051883	-0.42306	-0.40243	-0.56923	0.089	0.395	0.378	0.075	0.157	0.348	0.391	0.19
PC 34:2 (PC)	Phosphatidylcholine 34:2	LIPID282	0.208464	-0.50574	0.03381	-0.52972	0.140465	-0.43847	-0.39634	-0.5959	0.089	0.465	0.303	0.075	0.172	0.407	0.469	0.193
SM 38:1;2 (SM)	Sphingomyelin 38:1;2	LIPID286	0.223527	-0.53413	0.138705	-0.546	-0.00438	-0.46636	-0.40343	-0.60179	0.162	0.5	0.303	0.107	0.16	0.456	0.393	0.226
PC 34:1 (PC 104)	Phosphatidylcholine 34:1	LIPID288	0.193031	-0.54018	0.145841	-0.33417	-0.06581	-0.44541	-0.32483	-0.68456	0.088	0.329	0.378	0.107	0.219	0.475	0.373	0.336
PC 34:1 (LPC)	Lyso-Phosphatidylcholine 34:1	LIPID289	0.17285	-0.55912	-0.00511	-0.51677	-0.05861	-0.48577	-0.15543	-0.61666	0.273	0.465	0.303	0.107	0.405	0.401	0.386	0.445
PC 34:1 (PC)	Phosphatidylcholine 34:1	LIPID291	0.147259	-0.54657	-0.01343	-0.59329	0.002898	-0.48588	-0.1656	-0.60383	0.273	0.5	0.459	0.208	0.405	0.408	0.427	0.408
SM 38:0;2 (SM)	Sphingomyelin 38:0;2	LIPID294	0.14536	-0.51834	0.312107	-0.46989	-0.16462	-0.49389	-0.35484	-0.53209	0.089	0.215	0.181	0.075	0.14	0.419	0.346	0.308
PC 34:0 (PC 104)	Phosphatidylcholine 34:0	LIPID298	0.135548	-0.61791	-0.30945	-0.61791	0.482962	-0.09319	-0.23958	-0.61791	0.454	0.459	0.363	0.349	0.429	0.23	0.349	0.187
PC 34:0 (LPC)	Lyso-Phosphatidylcholine 34:0	LIPID299	0.20471	-0.50275	0.027668	-0.48943	0.231552	-0.43998	-0.45856	-0.59024	0.162	0.5	0.378	0.075	0.134	0.399	0.488	0.16
PC 34:0 (PC)	Phosphatidylcholine 34:0	LIPID301	0.188208	-0.54708	0.099959	-0.59774	0.133556	-0.48977	-0.43265	-0.60129	0.42	0.329	0.303	0.208	0.153	0.451	0.474	0.189
SM 36:1;4 (SM)	Sphingomyelin 36:1;4	LIPID304	0.042366	-0.50798	0.492191	-0.17891	-0.36275	-0.45978	-0.25283	-0.47507	0.12	0.096	0.454	0.451	0.16	0.296	0.262	0.324
SM 40:4;2 (SM)	Sphingomyelin 40:4;2	LIPID345	0.180681	-0.49851	-0.03742	-0.4647	0.19113	-0.47001	-0.3323	-0.56969	0.213	0.329	0.303	0.075	0.243	0.377	0.495	0.228
PC 36:4 (LPC)	Lyso-Phosphatidylcholine 36:4	LIPID350	0.24782	-0.43459	-0.09886	-0.49863	0.187069	-0.47109	-0.34818	-0.44331	0.42	0.5	0.5	0.453	0.225	0.315	0.474	0.208
PC 36:4 (PC)	Phosphatidylcholine 36:4	LIPID352	0.190162	-0.48028	-0.03927	-0.48001	0.231876	-0.47727	-0.37442	-0.55102	0.121	0.464	0.378	0.075	0.199	0.369	0.482	0.198
SM 40:3;2 (SM)	Sphingomyelin 40:3;2	LIPID356	0.331671	-0.45494	-0.228	-0.44351	0.156656	-0.42237	-0.29533	-0.48816	0.162	0.5	0.181	0.075	0.4	0.228	0.427	0.222
PC 36:3 (LPC)	Lyso-Phosphatidylcholine 36:3	LIPID360	0.30875	-0.44749	-0.15573	-0.32579	0.10427	-0.37609	-0.29818	-0.50951	0.162	0.465	0.237	0.075	0.332	0.275	0.414	0.23
PC 36:3 (PC)	Phosphatidylcholine 36:3	LIPID362	0.266816	-0.49608	-0.15284	-0.50408	0.203778	-0.43873	-0.33037	-0.51835	0.213	0.465	0.181	0.107	0.296	0.279	0.473	0.21
SM 40:2;2 (SM)	Sphingomyelin 40:2;2	LIPID368	0.404392	-0.49908	-0.14972	-0.44051	0.015657	-0.41906	-0.34807	-0.55158	0.121	0.465	0.237	0.075	0.263	0.241	0.338	0.229
PC 36:2 (LPC)	Lyso-Phosphatidylcholine 36:2	LIPID371	0.24371	-0.46757	-0.01178	-0.48338	0.19063	-0.42969	-0.43318	-0.66469	0.162	0.395	0.303	0.107	0.167	0.359	0.476	0.175
PC 36:2 (PC)	Phosphatidylcholine 36:2	LIPID373	0.309857	-0.54925	-0.07334	-0.51926	0.07119	-0.41841	-0.35544	-0.56511	0.343	0.5	0.237	0.107	0.248	0.308	0.396	0.225
SM 40:1;2 (SM)	Sphingomyelin 40:1;2	LIPID377	0.386545	-0.51973	0.106745	-0.07271	-0.15358	-0.2583	-0.44773	-0.60927	0.121	0.464	0.378	0.107	0.139	0.368	0.26	0.144
PC 36:1 (LPC)	Lyso-Phosphatidylcholine 36:1	LIPID382	-0.01789	-0.61451	0.353824	-0.05144	0.128764	-0.3715	-0.43536	-0.65073	0.089	0.213	0.178	0.208	0.097	0.299	0.426	0.198
PC 36:1 (PC)	Phosphatidylcholine 36:1	LIPID383	0.202126	-0.62175	0.254327	-0.26099	-0.05049	-0.56688	-0.45648	-0.63248	0.089	0.327	0.458	0.107	0.098	0.472	0.38	0.228
SM 40:0;2 (SM)	Sphingomyelin 40:0;2	LIPID384	0.171018	-0.58708	0.106551	-0.16807	-0.14216	-0.27173	-0.19805	-0.17701	0.343	0.096	0.223	0.5	0.176	0.47	0.372	0.416
PC 38:6 (LPC)	Lyso-Phosphatidylcholine 38:6	LIPID406	0.448125	-0.31542	-0.20231	-0.30513	2.01E-05	-0.46003	-0.33546	-0.56941	0.26	0.39	0.456	0.349	0.344	0.199	0.313	0.262
PC 38:6 (PC)	Phosphatidylcholine 38:6	LIPID407	0.272592	-0.53369	-0.0753	-0.47232	0.057985	-0.46627	-0.2982	-0.52749	0.338	0.5	0.5	0.451	0.328	0.321	0.402	0.279
PC 38:5 (PC)	Phosphatidylcholine 38:5	LIPID415	0.297315	-0.54107	-0.10666	-0.5214	0.100962	-0.3604	-0.33089	-0.49461	0.419	0.428	0.335	0.275	0.313	0.299	0.412	0.222
SM 42:4;2 (SM)	Sphingomyelin 42:4;2	LIPID419	0.148933	-0.64775	0.140883	-0.23327	0.03603	-0.38884	-0.34843	-0.66148	0.213	0.213	0.335	0.279	0.175	0.496	0.448	0.265
PC 38:4 (LPC)	Lyso-Phosphatidylcholine 38:4	LIPID423	0.407601	-0.53455	-0.11443	-0.65549	0.071601	-0.18548	-0.43197	-0.66133	0.26	0.463	0.301	0.072	0.223	0.257	0.351	0.125
PC 38:4 (PC)	Phosphatidylcholine 38:4	LIPID425	0.368003	-0.56861	-0.08074	-0.54517	0.063138	-0.31595	-0.41138	-0.62288	0.159	0.5	0.301	0.074	0.2	0.284	0.368	0.164
SM 42:3;2 (SM)	Sphingomyelin 42:3;2	LIPID429	0.206618	-0.6028	0.324667	0.345392	-0.00035	0.00957	-0.57233	-0.72268	0.086	0.358	0.415	0.204	0.061	0.441	0.397	0.07
PC 38:3 (LPC)	Lyso-Phosphatidylcholine 38:3	LIPID434	0.259755	-0.58545	0.104726	-0.18482	-0.06346	0.063265	-0.36566	-0.5314	0.338	0.463	0.415	0.275	0.192	0.426	0.35	0.204
PC 38:3 (PC)	Phosphatidylcholine 38:3	LIPID435	0.262701	-0.65654	0.067249	-0.30698	0.095973	0.210708	-0.45927	-0.62224	0.38	0.428	0.415	0.177	0.163	0.406	0.42	0.08

			A (N=6)		AS (N=5)		H (N=4)		HS (N=5)		Wilcoxon Rank Test				T-test			
**Annotation**	**Expanded Annotation**	**LIPIDID**	**Mean**	**Median**	**Mean**	**Median**	**Mean**	**Median**	**Mean**	**Median**	**AS vs HS**	**A vs AS**	**A vs H**	**H vs. HS**	**AS vs HS**	**A vs AS**	**A vs H**	**H vs. HS**
SM 42:2;2 (SM)	Sphingomyelin 42:2;2	LIPID437	0.203391	-0.57914	0.162372	-0.10188	0.034839	0.175017	-0.43431	-0.68949	0.21	0.358	0.301	0.107	0.176	0.48	0.412	0.087
PC 38:2 (PC 104)	Phosphatidylcholine 38:2	LIPID439	-0.3985	-0.7071	0.166691	-0.7071	0.397913	0.365494	-0.00682	-0.7071	0.454	0.379	0.121	0.311	0.415	0.199	0.078	0.287
PC 38:2 (LPC)	Lyso-Phosphatidylcholine 38:2	LIPID440	0.015894	-0.50824	0.474515	0.112931	-0.27108	-0.119	-0.27672	-0.45756	0.307	0.294	0.415	0.318	0.168	0.285	0.316	0.494
PC 38:2 (PC)	Phosphatidylcholine 38:2	LIPID442	0.132086	-0.60483	0.278695	-0.28357	-0.00068	0.056177	-0.43666	-0.64882	0.121	0.213	0.415	0.241	0.157	0.429	0.424	0.132
SM 42:1;2 (SM)	Sphingomyelin 42:1;2	LIPID445	0.188176	-0.52915	0.293452	-0.24363	-0.06514	0.150488	-0.46715	-0.66107	0.273	0.213	0.415	0.318	0.138	0.449	0.359	0.109
Cer 30:0;4 (LCB 18:1;2-2H2O,LCB 18:0;3-3H2O)	Ceramide 30:0;4	LIPID54	0.238289	-0.63914	-0.38533	-0.63914	0.764119	0.877535	-0.51191	-0.63914	0.366	0.459	0.292	0.082	0.256	0.181	0.275	0.02
GM3 28:3;2 (LCB 18:1;2-2H2O,LCB 18:0;3-3H2O)	Monosialoganglioside 28:3;2	LIPID58	0.475274	0.593479	-0.5914	-0.54658	0.908965	0.889236	-0.7061	-0.68139	0.271	0.126	0.303	0.024	0.233	0.05	0.279	0.001
GM3 28:3;2 (LCB 18:1;2-H2O,LCB 18:0;3-2H2O)	Monosialoganglioside 28:3;2	LIPID59	0.424552	0.400448	-0.61264	-0.58951	0.939582	0.902401	-0.64848	-0.64529	0.5	0.126	0.303	0.024	0.419	0.059	0.246	0.001
CerP 28:1;2 (LCB 18:1;2-2H2O,LCB 18:0;3-3H2O)	Ceramide-phosphate 28:1;2	LIPID61	0.584071	0.628632	-0.66189	-0.66265	0.93041	0.940448	-0.78332	-0.75562	0.341	0.055	0.237	0.024	0.291	0.025	0.295	0
CerP 28:1;2 (LCB 18:1;2-H2O,LCB 18:0;3-2H2O)	Ceramide-phosphate 28:1;2	LIPID63	0.552456	0.612457	-0.64766	-0.6604	0.890288	0.968685	-0.72752	-0.6788	0.419	0.055	0.237	0.024	0.37	0.035	0.309	0
GM3 28:1;2 (LCB 18:1;2-2H2O,LCB 18:0;3-3H2O)	Monosialoganglioside 28:1;2	LIPID65	-0.15998	-0.48371	-0.12913	-0.05675	0.021285	-0.15505	0.304076	-0.29215	0.417	0.321	0.263	0.275	0.305	0.472	0.366	0.386
GM3 28:1;2 (LCB 18:0;2-2H2O)	Monosialoganglioside 28:1;2	LIPID66	0.393734	0.728264	-0.55939	-0.67503	0.786542	1.073412	-0.54233	-0.33387	0.458	0.126	0.337	0.103	0.472	0.05	0.312	0.034
GM3 28:1;2 (LCB 18:1;2-H2O,LCB 18:0;3-2H2O)	Monosialoganglioside 28:1;2	LIPID67	0.116019	-0.56524	-0.37758	-0.31347	0.200584	0.268279	0.077889	-0.15603	0.341	0.463	0.5	0.5	0.223	0.229	0.458	0.435
M(IP)2C 26:1;2 (LCB 18:1;2-2H2O,LCB 18:0;3-3H2O)	Mannosyl-diinositol Phosphate ceramide 26:1;2	LIPID69	-0.25261	-0.78557	0.546063	0.494921	-0.25783	-0.29172	-0.03667	-0.78557	0.333	0.196	0.5	0.448	0.239	0.134	0.496	0.362
CerP 28:0;2 (LCB 18:0;2-2H2O)	Ceramide-phosphate 28:0;2	LIPID70	0.341872	0.341053	-0.31474	-0.07634	0.677988	0.284574	-0.6379	-0.79622	0.147	0.126	0.5	0.082	0.098	0.109	0.346	0.054
M(IP)2C 26:1;2 (LCB 18:1;2-H2O,LCB 18:0;3-2H2O)	Mannosyl-diinositol Phosphate ceramide 26:1;2	LIPID71	-0.0825	-0.24741	0.175888	0.428131	0.294336	-0.44181	-0.31236	-0.81162	0.26	0.325	0.5	0.311	0.197	0.317	0.324	0.249
M(IP)2C 26:2;4 (LCB 18:1;2-2H2O,LCB 18:0;3-3H2O)	Mannosyl-diinositol Phosphate ceramide 26:2;4	LIPID82	0.52825	0.49386	-0.68891	-0.6959	0.926985	1.064152	-0.68658	-0.70388	0.5	0.074	0.378	0.024	0.489	0.028	0.288	0.001
M(IP)2C 26:2;4 (LCB 18:1;2-H2O,LCB 18:0;3-2H2O)	Mannosyl-diinositol Phosphate ceramide 26:2;4	LIPID83	0.511703	0.407853	-0.668	-0.67183	0.878144	0.975846	-0.64856	-0.67349	0.5	0.074	0.237	0.024	0.401	0.037	0.312	0.001
Cer 34:2;3 (LCB 18:2;2-2H20,LCB 18:1;3-3H2O)	Ceramide 34:2;3	LIPID86	0.840357	0.415264	-0.7136	-0.60745	0.681048	0.720745	-0.83967	-1.18353	0.417	0.012	0.378	0.023	0.316	0.003	0.387	0.002
CerP 28:0;3 (LCB 18:0;2-2H2O)	Ceramide-phosphate 28:0;3	LIPID87	0.246097	0.710426	-0.58195	-1.10004	0.819234	0.728175	-0.36876	-1.10004	0.454	0.156	0.23	0.095	0.36	0.097	0.185	0.038
CerP 28:0;3 (LCB 18:1;2-H2O,LCB 18:0;3-2H2O)	Ceramide-phosphate 28:0;3	LIPID88	0.567665	0.612801	-0.71717	-0.70884	0.974409	1.00462	-0.74356	-0.73968	0.42	0.042	0.378	0.024	0.42	0.019	0.268	0
M(IP)2C 26:0;4 (LCB 18:1;2-2H2O,LCB 18:0;3-3H2O)	Mannosyl-diinositol Phosphate ceramide 26:0;4	LIPID89	0.445933	0.160909	-0.51846	-0.8135	0.257923	0.239637	-0.223	-0.4961	0.419	0.126	0.5	0.151	0.267	0.093	0.408	0.207
M(IP)2C 26:0;4 (LCB 18:0;2-2H2O)	Mannosyl-diinositol Phosphate ceramide 26:0;4	LIPID90	0.469993	0.442493	-0.64777	-0.57109	0.848298	0.888239	-0.59486	-0.62972	0.419	0.126	0.459	0.024	0.345	0.05	0.312	0.002
M(IP)2C 26:0;4 (LCB 18:1;2-H2O,LCB 18:0;3-2H2O)	Mannosyl-diinositol Phosphate ceramide 26:0;4	LIPID91	0.392839	-0.38748	-0.25709	-0.6227	-0.17164	-0.2603	-0.077	-0.18418	0.341	0.463	0.456	0.452	0.35	0.204	0.26	0.427
Hex3Cer 40:1;2 (LCB 18:0;2-2H2O)	Trihexosylceramide 40:1;2	LIPID96	0.512331	0.6683	-0.52396	-0.46642	0.7218	0.798744	-0.66828	-0.65747	0.271	0.126	0.458	0.127	0.175	0.043	0.396	0.019
Hex3Cer 40:1;2 (LCB 18:0;2-H2O)	Trihexosylceramide 40:1;2	LIPID98	0.505772	0.575519	-0.60055	-0.49097	0.631701	0.903898	-0.51173	-0.35148	0.268	0.126	0.458	0.126	0.311	0.046	0.439	0.032
